# Cancer therapy-related cardiovascular aging: mechanisms, monitoring, and intervention strategies

**DOI:** 10.3389/fcvm.2025.1668623

**Published:** 2026-01-08

**Authors:** Xinyue Lin, Zhebin Wu, Yanfang Liu, Xiang Liao

**Affiliations:** 1 Institute of Medical Imaging and Artificial Intelligence, School of Medicine, Jiangsu University, Zhenjiang, China; 2 Department of Ultrasonic Diagnosis, Jingjiang People's Hospital, Jingjiang, Jiangsu Province, China; 3 Department of Central Laboratory, Affiliated People’s Hospital of Jiangsu University, Zhenjiang, China; 4 Department of Laboratory Medicine, Affiliated Hospital of Jiangsu University, Zhenjiang, China

**Keywords:** cancer therapy, cardiovascular aging, cellular senescence, mechanism, monitoring

## Abstract

Significant improvements in cancer survival rates have been achieved through advancements in treatment and early diagnosis. However, non-cancer-related mortality among cancer survivors continues to rise each year. Cardiovascular diseases (CVDs) related to cancer therapy now rank as the second leading cause of death in survivors, sometimes surpassing the cancer itself. Among these, cardiovascular aging represents one of the most severe side effects, often leading to detrimental structural changes such as cardiac atrophy or fibrosis, which ultimately impair cardiac function and reduce survival. Preventing or treating cardiovascular aging has emerged as a promising strategy to mitigate Cancer Therapy-Related Cardiovascular Toxicity (CTR-CVT). This review offers a comprehensive analysis of the characteristics and mechanisms underlying cancer therapy-induced accelerated cardiovascular cellular senescence, outlines current monitoring and intervention strategies, and explores future research opportunities and challenges. Enhancing the understanding of Cancer Therapy-Related Cardiovascular Cellular Senescence and Cancer Therapy-Related Cardiovascular Aging (CTR-CVA) is crucial for optimizing cancer treatment, advancing medical research, and improving clinical practice, all of which are vital for preserving cardiac health and improving the quality of life of patients with cancers.

## 1 Introduction

Advancements in treatment modalities and early precision diagnosis have significantly improved the survival rates of patients with cancers. According to the Cancer Research Institute, the number of cancer survivors in the United States grew from 18 million in 2022 to nearly 26 million by 2024 (1). Despite these gains, long-term survivors face the challenge of cancer treatment-related diseases, particularly cardiovascular diseases (CVDs). Cancer Therapy-Related Cardiovascular Toxicity (CTR-CVT), referring to a series of acute and chronic cardiovascular system injuries occurring after cancer treatment, has now emerged as the second leading cause of long-term morbidity and mortality among cancer survivors (2). Childhood cancer survivors (CCS) underwent chest radiotherapy are at a 10.6-fold increased risk of myocardial infarction and congestive heart failure later in life compared to their siblings (3). Consequently, exploration of the long-term underlying pathophysiological mechanisms, early prediction, diagnosis, and treatment of CTR-CVT remain key research priorities in the field of CTR-CVT (4). The establishment Cardio-Oncology marked the inception of this specialized discipline (5). Recent research has highlighted cellular senescence as a critical driver of Cancer Therapy-Related Cardiovascular Diseases (CTR-CVD). Senescent cardiovascular cells contribute to the remodeling of the extracellular matrix (ECM), disrupting normal cellular function and development, thereby increasing disease susceptibility (6, 7). This, in turn, elevates the risk of major adverse cardiovascular events (MACE) in the patients with cancers (7).

**Figure 1:**
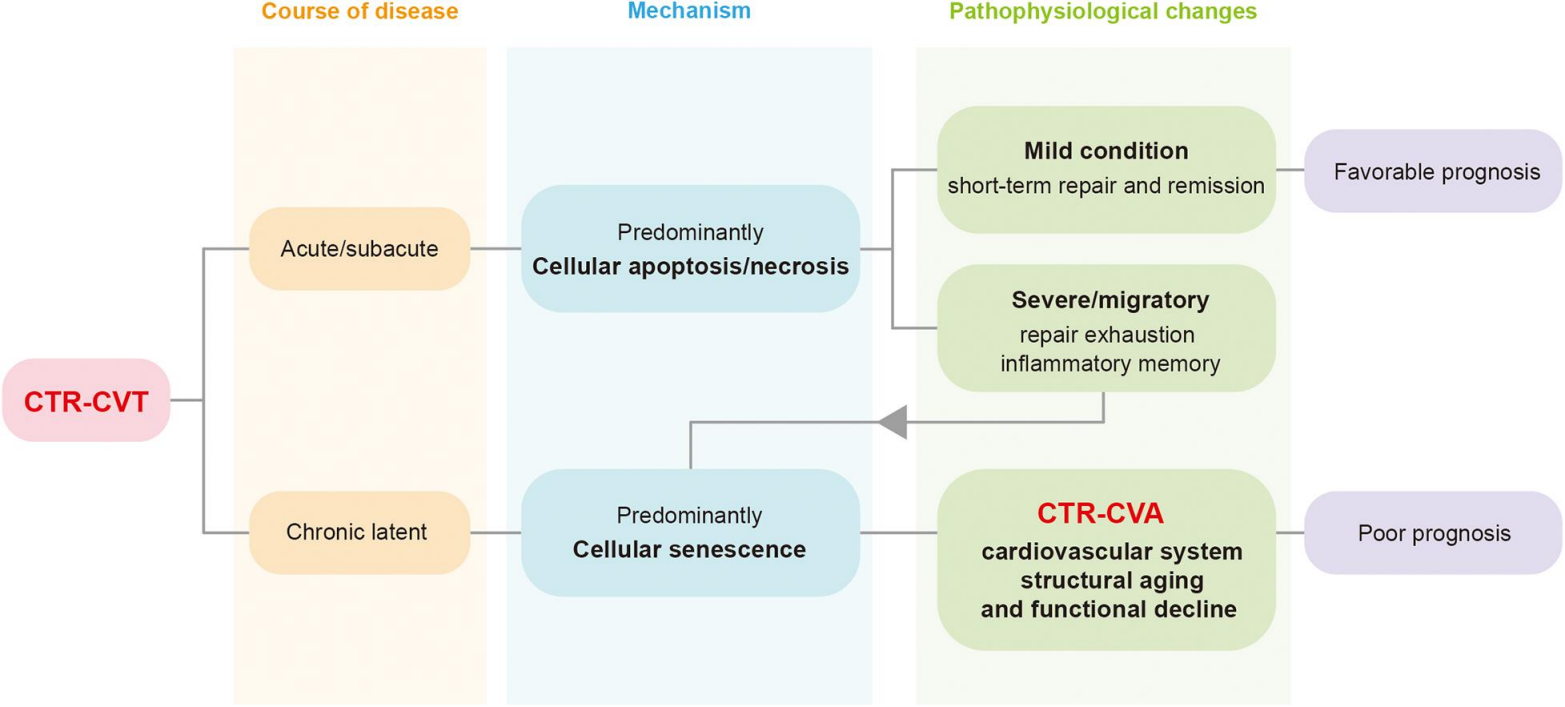
The relationship between Cancer Therapy-Related Cardiovascular Toxicity (CTR-CVT) and Cancer Therapy-Related Cardiovascular Aging (CTR-CVA). CTR-CVT is a collection of a series of acute and chronic injuries after cancer treatment. CTR-CVA essentially represents the long-term progression of acute/subacute injuries and the chronic phenotype of latent injuries in CTR-CVT. CTR-CVA is characterized by the accumulation of senescent cells, manifested as structural degeneration and functional decline of the cardiovascular system. CTR-CVT, Cancer Therapy-Related Cardiovascular Toxicity; CTR-CVA, Cancer Therapy-Related Cardiovascular Aging.

Aging is a process in which the overall functions of an organism gradually decline over time. As a key intrinsic driving mechanism underlying macroscopic aging, cellular senescence leads to the functional decline of tissues and organs and promotes the continuous progression of the aging process by inducing cell cycle arrest and senescence-associated metabolic changes. Cardiovascular aging is a physiological or pathological process characterized by structural degradation and functional decline of the cardiovascular system, with cellular senescence as its fundamental cytological basis, occurring under the influence of aging or pathological factors. With the continuous expansion and in-depth development of relevant research, the academic view that the arrest of the cellular senescence cycle is irreversible is gradually being questioned and revised (8–10), opening up a breakthrough for the intervention in cell senescence-related diseases. In recent years, the American Institute for Cancer Research has begun to pay attention to the potential value of eliminating senescent cells, including those in normal tissues (11). Previous articles have put forward the concept of the Aging-Cancer Cycle, suggesting that there is a two-way relationship between cancer treatment and aging (12). In 2024, articles in the JACC series proposed the existence of cardiovascular aging under tumor treatment (13). This type of cardiovascular aging, caused by the continuous accumulation of senescent cells in the cardiovascular system induced by cancer therapy, can be defined as Cancer Therapy-Related Cardiovascular Aging (CTR-CVA). It represents the long-term progression of acute or subacute injuries in CTR-CVT, as well as a branching manifestation of chronic injuries in CTR-CVT (Figure 1).

**Figure 2:**
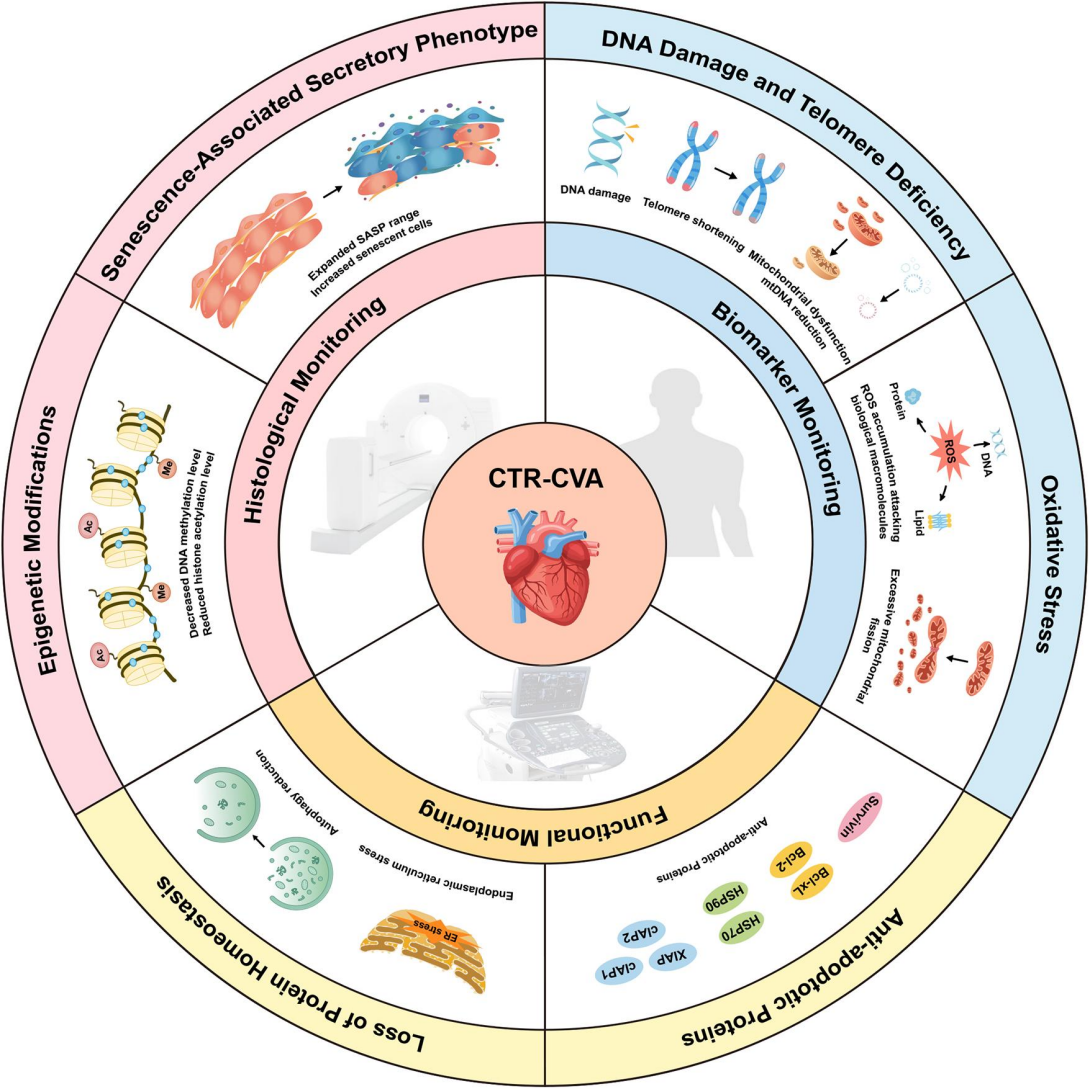
Mechanisms and Monitoring of Cancer Therapy-Related Cardiovascular Aging (CTR-CVA). CTR-CVA is based on cellular senescence and involves six mechanisms: DNA damage and telomere deficiency, oxidative stress, upregulation of anti-apoptotic proteins, disruption of protein homeostasis, epigenetic modifications, and the senescence-associated secretory phenotype. In this review, the monitoring of CTR-CVA is categorized into biomarker-based monitoring, functional monitoring, and histological monitoring. CTR-CVA, Cancer Therapy-Related Cardiovascular Aging; ER, endoplasmic reticulum; mtDNA, mitochondrial DNA; SASP, senescence-associated secretory phenotype.

This review focuses on exploring the mechanisms of CTR-CVA, advancements in detecting and monitoring cardiovascular aging, and potential directions for future research. This article updates the existing reviews in this field, with a focus on the advanced imaging techniques currently used for identifying senescent cells and the popular therapeutic approaches. Strengthening interdisciplinary collaboration across Cardio-Oncology, materials science, molecular imaging, and artificial intelligence is crucial to support early diagnosis and intervention for CTR-CVT. In particular, effective treatments for cellular senescence could significantly improve the quality of life for cancer survivors (Figure 2).

## 2 Cancer therapy-related cardiovascular disease and cardiovascular aging

With the evolution of Cardio-Oncology, there is a growing understanding of the complexities surrounding CTR-CVD, leading to the development of comprehensive strategies for their diagnosis, prevention, therapy, and management (14). CTR-CVD encompasses a broad spectrum of diseases, including heart failure, arrhythmia, coronary artery disease (CAD), pericardial diseases, and valvular disorders (15, 16). Several factors influence the incidence of CTR-CVD: (i) Treatment modalities: patients treated with anthracyclines often experience severe cardiac adverse events, while liposomal doxorubicin (DOX) treatment tends to have fewer cardiac complications (17–19). (ii) Therapy-related drug accumulation and dosage cycles (20, 21): High-dose DOX is primarily associated with acute declines in cardiac function or an increased incidence of arrhythmias. In contrast, low-dose DOX is linked to long-term, atypical cardiac symptoms or cardiovascular dysfunction (22). The risk of heart failure increases with cumulative DOX dosage, with incidences of 5%, 26%, and 48% when the cumulative dose reaches 400 mg/m^2^, 550 mg/m^2^, and 700 mg/m^2^, respectively, with most cases manifesting within the first year of treatment (23). This study supplied the evidence that the cumulative DOX dose should not exceed 500–550 mg/m^2^ in clinical trials (20, 21). (iii) Early and delayed side effects: The onset and pattern of CTR-CVD symptoms varied according to the cancer treatment. Radiation-induced pericarditis typically occurs shortly after exposure, while other radiation-related heart diseases often emerge 10–15 years later (24). (iv) Individual differences: Even under the same therapeutic regimen, individuals may experience distinct manifestations. Elderly patients, particularly those undergoing combination therapies, are more prone to aging-related phenomena such as palpitations, fatigue, and elevated serum biomarkers indicative of injury (7). Epidemiological studies have shown that childhood cancer survivors experience a significantly higher incidence of severe cardiovascular adverse events compared to their siblings (25). Imaging and pathological assessments of these survivors reveal alterations in cardiovascular structure and function, including ventricular remodeling, myocardial fibrosis, and valvular calcification (26).

Cellular senescence is a key factor in the onset, progression, and outcomes of various diseases, influencing the entire pathophysiological process (27). Various cancer treatments can induce celluar senescence in healthy, non-cancerous cells. The accumulation of senescent cells plays a central role in driving premature tissue aging. In CTR-CVD, the importance of cardiovascular aging is increasingly recognized by researchers. Cardiovascular aging and cellular senescence can be detected in the peripheral blood (28), cardiac electrophysiology (29), or tissue specimens (30) of patients with cancers long after treatment (Table 1). Similar to natural aging, CTR-CVA can trigger tissue remodeling phenomena such as vascular wall thickening and cardiac fibrosis, accompanied by functional deterioration manifestations such as increased arterial stiffness and decreased cardiac diastolic function. Although some pathological features and changes in clinical indicators are similar to those of natural cardiovascular aging, CTR-CVA is often more severe. The essential difference between the two is that natural aging is a slow and progressive process that follows biological laws, while cancer treatments such as radiotherapy and chemotherapy can activate cellular senescence and related signaling pathways earlier through exogenous stimulation, thereby accelerating the process of cardiovascular aging. Therefore, Cardiovascular cellular senescence has become a key link in the pathogenesis of CTR-CVD (31), a comprehensive understanding of its mechanisms is essential for the prevention and treatment of CTR-CVT.

**Table 1:** Clinical evidence associated with cancer therapy-related cardiovascular aging.

Sample types	Grouping of research subjects	Evidence of aging	Specific indicators	Literature results in detail	Year
Patient Cardiac Specimens	Treatment group: Autopsy cardiac specimens of 6 tumor patients who had received DOX treatment and died of congestive heart failure; Control group: Autopsy cardiac specimens of 6 patients with non-cardiovascular causes of death	Cell cycle arrest and cardiac fibrosis	p16^INK4a^, γ-H2AX, Masson trichrome staining area	In the cardiac progenitor cells (CPCs) of the cardiomyopathy cardiac specimens exposed to DOX treatment, the expressions of p16^INK4a^ and γ-H2AX increased	2013 (30)
Patient Blood Specimens	10 blood samples at different time points from patients receiving combination chemotherapy of DOX and cyclophosphamide	Decrease in circulating endothelial progenitor cells (CEPs)	CEPs positive for CD-133 and Vascular Endothelial Growth Factor Receptor-2 (VEGFR-2)	In patients exposed to DOX and cyclophosphamide, the number of serum CD-133 and VEGFR-2 positive CEPs showed a linear downward trend from baseline to the 12th week	2013 (32)
	Cross-sectional survivor cohort: 176 female survivors aged 50 and above who had undergone surgical resection for stage I-III breast cancer, with 69 receiving adjuvant chemotherapy and the rest not; Prospective adjuvant treatment cohort: 33 female patients diagnosed with stage I-III breast cancer and planned to receive adjuvant chemotherapy	Cell cycle arrest and increased SASP expression	p16^INK4a^ and Alternative Reading Frame (ARF), Interleukin-6 (IL-6), Interleukin-7 (IL-7), Interleukin-8 (IL-8), Vascular Endothelial Growth Factor A (VEGFA), and Monocyte Chemoattractant Protein-1 (MCP1)	In the peripheral blood T lymphocytes and serum samples of breast cancer survivors who had received adjuvant chemotherapy, the expressions of p16^INK4a^ and ARF increased, and the expressions of multiple SASP factors in the serum were significantly higher than those in patients who had not received adjuvant chemotherapy	2,014 (33)
	Treatment group: 87 asymptomatic childhood acute lymphoblastic leukemia (ALL) survivors, of which 48.3% had received combined treatment of chemotherapy and radiotherapy, and the rest had only received chemotherapy; Control group: 87 healthy volunteers	Telomere shortening and increased SASP expression	Telomere length, Interleukin-2 (IL-2), Interleukin-10 (IL-10), Interleukin-17a (IL-17a), High-sensitivity C-reactive protein (hsCRP)	The peripheral blood leukocyte telomere length of ALL survivors who had received chemotherapy or combined treatment of chemotherapy and radiotherapy was significantly shorter than that of the control group, and the levels of multiple inflammatory factors in the plasma increased. Among them, the telomeres of patients who had received radiotherapy were shorter than those of survivors who had only received chemotherapy	2017 (34)
	15 post-menopausal female breast cancer survivors, Non-lateralized cerebral oxygenation group: The difference in cerebral oxygenation between the left and right hemispheres was less than 3%; Lateralized cerebral oxygenation group: The difference in cerebral oxygenation between the left and right hemispheres was ≥ 3%	Decline in cerebral cognitive function and increased SASP expression	IL-6, Tumor Necrosis Factor α (TNF-α), CRP, and Insulinlike Growth Factor-1 (IGF-1)	The increase in the levels of SASP and other factors and the decrease in the level of IGF-1 in the peripheral blood samples of breast cancer patients who had received chemotherapy indicated accelerated cerebrovascular aging, which led to the decline in cerebral cognition	2018 (35)
	Treatment group: 72 newly-diagnosed stage 0-IIIA breast cancer patients after treatment, of which 35 had received combined treatment of chemotherapy and radiotherapy, and the remaining 37 had only received radiotherapy; Control group: The above 72 newly-diagnosed stage 0-IIIA breast cancer patients before treatment	DNA methylation and epigenetic changes	Extrinsic Epigenetic Age Acceleration (EEAA), Phenotypic Epigenetic Age Acceleration (PEAA), and Grim Epigenetic Age Acceleration (GEAA)	In the DNA methylation detection results of the peripheral whole blood samples of breast cancer patients after radiotherapy and chemotherapy, it was observed that indicators such as EEAA, PEAA, and GEAA all increased	2020 (28)
	55 blood samples from patients with metastatic testicular cancer before and after treatment with cisplatin	Telomere shortening	Telomere length	The peripheral blood leukocyte telomeres of 7/55 patients with metastatic testicular cancer shortened one year after cisplatin treatment	2024 (36)
Patient Electrocardiogram Analysis	Group1: 15 and 22 childhood survivors who had only received anthracycline-based treatment of ≥300 mg/m^2^ and <300 mg/m^2^ respectively; Group2: 15 and 22 childhood survivors who had received thoracic radiotherapy and anthracycline-based treatment of ≥300 mg/m^2^ and <300 mg/m^2^ respectively	Prolongation of QT interval	Corrected QT interval (QTc)	The incidence of QTc prolongation (≥ 0.43) in children cancer patients who received higher doses of anthracyclines was higher than that in the low-dose group, and chest radiotherapy may have a synergistic effect.	1993 (29)

## 3 Mechanisms of cancer therapy-related cardiovascular aging

**Figure 3:**
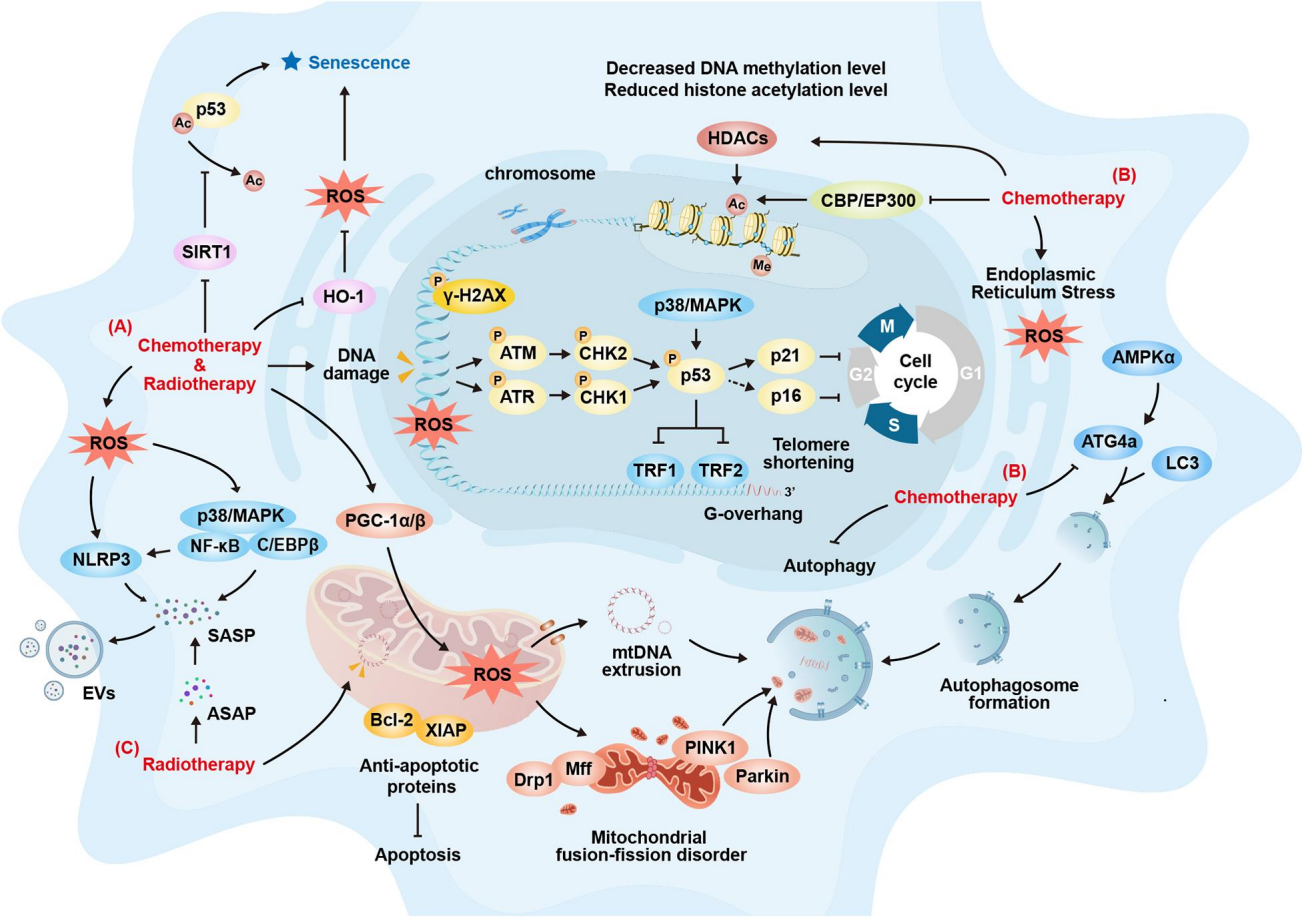
Cardiovascular Cellular Senescence Signaling during Chemotherapy and Radiotherapy. **(A)** Common Mechanisms of Cellular Senescence Induced by Chemotherapy and Radiotherapy: Both can induce DNA damage and abnormal repair, activating classical cellular senescence pathways such as the p53-p21 and p16-Rb pathways, which promotes cell-cycle arrest, leading cells to enter a state of cellular senescence; Both can induce oxidative stress related cellular senescence by activating the P38/MAPK-NFκB pathway or the NOD-like receptor family pyrin domain containing 3 (NLRP3) inflammasome; Both can result in the secretion of various cytokines through pro-inflammatory reactions, causing the accumulation and spread of the senescence-associated secretory phenotype (SASP), thus inducing cellular senescence in adjacent cells; Both can increase the expression of anti-apoptotic proteins such as Bcl-2 and X-linked inhibitor of apoptosis protein (XIAP) to counteract damage. Cells then enter a state of cellular senescence to avoid excessive apoptosis; Both can affect the mitochondrial oxidative respiratory chain, causing the accumulation of ROS within mitochondria, which exacerbates cellular senescence. **(B)** Other Mechanisms of Cellular Senescence Induced by Chemotherapy: Chemotherapy can promote telomere shortening and cellular senescence by downregulating the expression of telomeric repeat-binding factor 1 (TRF1) and telomeric repeat-binding factor 2 (TRF2); Chemotherapy can induce cellular senescence by promoting endoplasmic reticulum dysfunction, leading to protein homeostasis imbalance and exacerbating endoplasmic reticulum stress; Chemotherapy can promote cellular senescence by reducing DNA methylation; Chemotherapy can promote cellular senescence by upregulating histone deacetylases (HDACs), downregulating histone-acetylation-regulating genes such as CBP and Ep300, and thus decreasing the level of histone acetylation. It can also induce senescence by inhibiting SIRT1 to activate p53. **(C)** Other Mechanisms of Cellular Senescence Induced by Radiotherapy: Radiotherapy can impact mitochondrial DNA (mtDNA), affecting normal biosynthesis and promoting cellular senescence; Radiotherapy can trigger the release and maintenance of short-term acute-stress-associated phenotypes (ASAP). This prolongs the duration of inflammatory stimulation and gradually transforms it into SASP, promoting cellular senescence and its spread. ATG4a, autophagy-related gene 4a; ATM, ataxia-telangiectasia mutated; ASAP, acute stress-associated phenotype; DRP1, dynamin-related protein 1; EVs, extracellular vesicles; HDACs, histone deacetylases; HO-1, heme oxygenase-1; MFF, mitochondrial fission factor; mtDNA, mitochondrial DNA; NLRP3, NOD-like receptor family pyrin domain containing 3; PGC-1α/β, peroxisome proliferator-activated receptor gamma co-activator 1-Alpha/Beta; PINK1, PTEN-induced putative kinase 1; ROS, reactive oxygen species; SASP, senescence-associated secretory phenotype; SIRT1, sirtuin 1; TRF1/2, telomeric repeat-binding factor 1/2; XIAP, X-linked inhibitor of apoptosis protein.

Cellular senescence is the key underlying mechanism of tissue aging. Cellular senescence is characterized by a permanent proliferation arrest in response to both endogenous and exogenous stress or damage, triggering a range of intracellular phenotypic changes (37–39). The hallmarks of cellular senescence include cell cycle arrest, activation of the DNA damage response (DDR), nuclear alterations, upregulation of anti-apoptotic factors, the senescence-associated secretory phenotype (SASP), metabolic adaptation, morphological changes, increased lysosomal content, and the expression of specific cell surface markers (31, 39). Recent studies have also identified the accumulation of immunoglobulins as an important marker of senescence (40). Under prolonged stress or disease conditions, the continuous accumulation of senescent cells will lead to a decline in essential repair functions and immune clearance capabilities, alterations in intercellular communication, changes in the extracellular matrix (41), chronic inflammation, and so on. This will continuously accelerate the accumulation of cellular senescence and form a negative feedback loop, further exacerbating tissue aging. Baker et al. developed an “Inducible and Regulatable Targeted Apoptosis of Senescent Cells (INK-ATTAC)” system using transgenic technology, which targets cells expressing p16^INK4a^ (42). This technique allowed for the anatomical localization of senescent cells in the kidneys of aged mice and demonstrated significant efficacy in their targeted clearance. By eliminating senescent cardiac cells with AP20187, cardiomyocyte hypertrophy in aged mice was alleviated without compromising heart function (42). These findings provided valuable insight into the link between cellular senescence and cardiac aging. Similar studies lay the theoretical groundwork for the targeted clearance of senescent cells as a strategy to mitigate CTR-CVT (43). Therefore, the mechanisms underlying CTR-CVA, particularly those induced by conventional chemotherapy and radiotherapy, will be discussed in relation to markers of cellular senescence in the following sections (Figure 3).

### 3.1 Mechanisms of anthracycline-induced cardiovascular aging

Anthracyclines are first-line chemotherapy agents used to treat various malignancies, including breast cancer, lymphoma, and acute leukemia (44). DOX, epirubicin, and daunorubicin belong to the anthracycline. Among these, DOX is the earliest discovered and most widely used. Unfortunately, the cardiovascular adverse effects of DOX, a first-generation anthracycline, have been recognized in a dose-dependent manner since 1976 (45, 46). The molecular mechanisms underlying DOX-induced cardiac senescence are complex, involving DNA damage and telomere deficiency, oxidative stress, upregulation of anti-apoptotic proteins, loss of protein homeostasis, epigenetic modifications, and SASP (Table 2). It is important to note that in current cellular and animal experiments designed to establish stable cellular senescence models, the drug dosages employed in most studies—after relevant conversion—exceed the clinical threshold (<500–550 mg/m^2^). Consequently, the experimental results may not accurately recapitulate the authentic pathological conditions and underlying mechanisms of cardiovascular cellular senescence induced by cancer therapy in clinical practice.

**Table 2:** Mechanisms associated with cancer therapy-related cardiovascular aging.

Treatment modalities	Experimental subjects	Treatment protocols	Detection of senescence	Mechanisms	Year
Anthracycline Drugs	*In vitro*: Isolated neonatal rat cardiomyocytes	*In vitro*: Treatment with 10^−7^ mol/L DOX for 7 days	Increased SA-β-gal activity; Decreased in telomerase activity; Shortened telomere length; Increased protein levels of P27^kip1^ and p21^cip1/waf1^; Increased mRNA levels of p16^INK4a^	The activation of the PML-acetylated p53 complex	2008 (47)
	*In vivo*: 4-week-old male Wistar rats	*In vivo*: Intraperitoneal administration of DOX, divided into six equal injections (2.5 mg/kg each) over 2 weeks, with a total cumulative dose of 15 mg/kg body weight, with sample collection for monitoring at 11 months of age			
	*In vitro*: Isolated Ventricular Myocytes from 2-Day-Old Sprague-Dawley Rats (Lonza) and H9c2 Rat Cardiomyoblasts	*In vitro*: Treatment with 0.1 μM DOX for 3 h, followed by assessment at 24 and 48 h	Increased SA-β-gal activity; Cellular senescence morphology; Increased micronuclei; Chromosomal abnormalities; Increased protein levels of p53	The downregulation of TRF1 and TRF2 via MAPK and p53-mediated pathways	2009 (48)
	*In vitro*: Isolated c-kit positive human cardiac progenitor cells	*In vitro*: Treatment with 0.1, 0.5, and 1.0 μM DOX for 24 and 48 h, followed by 7 days of culture	Increased SA-β-gal activity; Increased tissue immunofluorescence labeling of p16^INK4a^ and γ-H2AX; Decreased expression of BrdU and Ki67; Activation of DNA damage pathways	The activation of the p16-Rb pathway-triggered cell cycle arrest	2013 (30)
	*In vivo*: Human cardiac autopsy specimens	*In vivo*: Standard clinical doses for cancer patients			
	*In vitro*: Human primary umbilical artery vascular smooth muscle cells (VSMCs) and mouse aortic VSMCs.	*In vitro*: Treatment with 0.25, 0.5, and 1 µM DOX for 3 h, followed by replacement with normal culture medium and monitoring after 3 days	Increased SA-β-Gal activity; Decreased cell viability; Increased protein levels of p53, p21^Cip1/Waf1^, and p16^INK4a^	The upregulation of uPAR-triggered TRF2 ubiquitination and proteasomal degradation	2013 (49)
	*In vitro*: Human vascular smooth muscle cells	*In vitro*: Treatment of passages 5–8 cells with 100 nM DOX to induce premature senescence, followed by analysis at 1, 3, and 7 days	Increased SA-β-gal activity; Cellular senescence morphology; Cell cycle arrest; Increased DNA damage-associated 53BP1 foci; Increased micronuclei formation; Increased γ-H2AX levels; Increased protein levels of Increased p21^cip1/waf1^ protein levels; Increased expression of SASP; Increased superoxide production; DNA methylation inhibition	The Activation of DNA Damage Repair-related ATM Pathway-coupled with Superoxide Level Accumulation	2014 (50)
	*In vitro*: H9c2 cells and isolated primary cardiomyocytes from mice.	*In vitro*: Pre-treatment with 0.1 μmol/L DOX for 3 h, followed by sample collection after 21 h, 45 h, 7 days, 14 days, and 21 days in normal culture medium	Increased SA-β-gal activity; Increased protein levels of p53, and p16^INK4a^	The downregulation of TRF2-induced reduction in telomere protection	2016 (51)
	*In vivo*: 4-week-old male Wistar rats	*In vivo*: Subcutaneous injection of DOX at 2 mg/kg once weekly for 7 weeks, total cumulative dose of 14 mg/kg, with sample collection in week 9	Reduced mitochondrial DNA (mtDNA); Decreased DNA methylation levels	The depletion of mtDNA-coupled with DNA methylation reduction	2017 (52)
	*In vitro*: HL-1 mouse cardiomyocytes	*In vitro*: Treatment with 5 µM DOX for 0, 24, 48, and 72 h	Decreased cell viability; Shortened telomere length; Decreased in telomerase activity; Elevated intracellular ROS; Elevated mitochondrial superoxide; Increased mRNA levels of p53, p27^Kip1^, and p16^INK4a^	The increase in lincRNA-p21 expression-coupled with Wnt/β-catenin Signaling Pathway Activation and ROS-related Indicator Increase	2018 (53)
	*In vitro*: H9c2 cells	*In vitro*: Treatment with 0.5 µM DOX for 72 h	Decreased cell viability; Increased mRNA levels of p53 and p16^INK4a^; Shortened telomere length; Decreased in telomerase activity	The Elevation of TGF-β1 Expression	2018 (54)
	*In vitro*: H9c2 rat embryonic cardiomyocytes	*In vitro*: Treatment with 0.1 µM DOX for 24 h	Increased SA-β-gal activity	The miR-34a/PNUTS axis	2019 (55)
	*In vitro*: H9c2 cells	*In vitro*: Treatment with 0.1 µM DOX for 48 h	Increased SA-β-gal activity; Decreased cell viability; Increased protein levels of p21^cip1/waf1^ and p16^INK4a^; Increased expression of SASP; Elevated intracellular ROS	The upregulation of TXNIP-triggering intracellular redox imbalance and NLRP3 inflammasome activation	2020 (56)
	*In vitro*: Isolated neonatal rat cardiomyocytes and H9c2 cells; *In vivo*: 3–4 week old Wistar rats	*In vitro*: Treatment with 50 nM DOX for 3 h, followed by monitoring after 7 days; *In vivo*: Treatment with DOX through intraperitoneal injections 9 times every other day at 5 mg/kg, with a total cumulative dose of 45 mg/kg, collect samples after 8 months	Increased SA-β-gal activity; Reduced mtDNA; Increased numbers of p16^INK4a^ positive cells and myosin positive cells under flow cytometry.	The damage of mtDNA	2020 (57)
	*In vitro*: Cardiomyocytes differentiated from human induced pluripotent stem cells (iPSC)	*In vitro*: Treatment with 0.5 μM DOX for 24 h	Increased SA-β-gal activity; Cell cycle arrest; Increased mRNA levels of p53 and p21^cip1/waf1^	The upregulation of miR-92a-3p and inhibition of ATG4a expression-induced attenuation of mitochondrial metabolism	2020 (58)
	*In vitro*: Isolated mouse ventricular myocytes; *In vivo*: eight-week-old male C57BL/6 mice	*In vitro*: Treatment with 1 μM DOX for 72 h; *In vivo*: Intraperitoneal injection of DOX was carried out on Mondays, Wednesdays and Fridays within one week, at a dose of 4 mg/kg each time, with a total cumulative dose of 12 mg/kg. Sample collection for monitoring was initiated on the 14th day	Increased SA-β-gal activity; Increased mRNA levels of p27^kip1^, p16^INK4a^, and p21^cip1/waf1^	The activation of the miR-221–3p/Sirt2 pathway	2020 (59)
	*In vitro*: Isolated neonatal mouse cardiomyocytes	*In vitro*: Treatment with 1 μM DOX for 24 h and 7 days	Increased SA-β-gal activity; Increased γ-H2AX foci; Decreased cell viability; Increased protein levels of p53 and p21^cip1/waf1^; Elevated mitochondrial ROS; Elevated intracellular ROS; Increased expression of SASP	The downregulation of mitochondrial autophagy via TBK1 K63-linked polyubiquitination facilitation	2021 (60)
	*In vitro*: H9c2 cells	*In vitro*: Treatment with 0.5 µM DOX for 24 h, followed by replacement with normal culture medium and monitoring after 10 days	Increased SA-β-gal activity; Decreased cell viability; Shortened telomere length; Increased intracellular ROS and mitochondrial superoxide; Increased mRNA levels of p53 and p21^cip1/waf1^	The depletion of Sirt6-caused mitochondrial damage, telomere dysfunction, increased H3K9 acetylation and upregulated NF-κB-related oxidative stress	2021 (61)
	*In vitro*: H9c2 cells; *In vivo*: C57BL/6 mice	*In vitro*: Pre-treatment with 0.1 µM DOX for 3 h, followed by replacement with normal culture medium and monitoring after 0, 24, 48 and 72 h; *In vivo*: Intraperitoneal injections at 2.5 mg/kg, three times a week for two consecutive weeks, with a cumulative dose of 15 mg/kg, collect samples for monitoring 4 months after completion of injections	Increased SA-β-gal activity; Decreased cell viability; Increased protein levels of p53, p21^Cip1/Waf1^, and p16^INK4a^; Increased expression of SASP	The p38 MAPK-Redd1-NF-κB pathway	2021 (62)
	*In vitro*: H9c2 rat embryonic cardiomyocytes and AC16 human cardiomyocyte-like cells	*In vitro*: Treatment with 0.1 μmol/l DOX for 24 h	Increased SA-β-gal activity; Shortened telomere length; Decreased in telomerase activity; Increased protein levels of p53, p21^cip1/waf1^, p16^INK4a^, and IGFBP3	The upregulation of C5a and C5aR-induced elevation of TNF-α and IFN-γ expression and ROS level	2021 (63)
	*In vivo*: Human ventricular cardiac fibroblasts (HCF), human umbilical vein endothelial cells (HUVECs); *In vitro*: 6-week-old wild-type BALB/c female mice	*In vivo*: Treatment with 100 nM DOX for 7 days, followed by replacement with serum-free medium and monitoring after 2 days; *In vitro*: Inject dox dissolved in 0.9% saline subcutaneously every other day starting on the 7th day after tumor formation, with a cumulative dose of 22 mg/kg, collect samples after 3 weeks	Increased protein levels of p21^cip1/waf1^ and p16^INK4a^	/	2022 (64)
	*In vitro*: H9c2 cells	*In vitro*: Pre-treatment with 0.3 μM DOX for 24 h	Increased SA-β-gal activity; Decreased cell viability; Shortened telomere length; Increased intracellular ROS and mitochondrial superoxide; Increased mRNA levels of p53 and p21^cip1/waf1^	The Klotho/SIRT1 signaling pathway.	2022 (65)
	*In vitro*: Immortalized human umbilical vein endothelial cell line EA.hy926 and primary HUVECs	*In vitro*: Treatment with 0.5µM DOX for 24 h, followed by replacement with normal culture medium and monitoring after 72 h and 5 days	Increased SA-β-gal activity; Cell cycle arrest; Increased protein levels of p53 and p21^cip1/waf1^; Increased expression of SASP;	The promotion of anti-apoptotic protein expression	2022 (66)
	*In vitro*: Cardiomyocytes differentiated from iPSC (iCM) and isolated mouse cardiomyocytes; *In vivo*: Cardiac tissue transcriptomics data	*In vitro*: Treatment with sub-lethal concentration of 0.2 μM DOX for 3 h, followed by replacement with normal culture medium and monitoring after 4 days	Increased SA-β-gal activity; Cell cycle arrest; Increased γ-H2AX positive nuclear foci; Increased mRNA and protein levels of p21^cip1/waf1^ and p16^INK4a^; Increased expression of SASP; Increased cell size; Elevated intracellular ROS	The loss of mitochondrial membrane potential-coupled with ROS increase	2022 (67)
	*In vitro*: HL-1 cardiomyocytes	*In vitro*: Treatment with 100 nM DOX for 72 h; *In vivo*: Administer a total cumulative dose of 20 mg/kg intraperitoneally in 8 injections over 4 weeks at 2.5 mg/kg per injection, and collect samples on the 44th day	Increased SA-β-gal activity; Cell cycle arrest; Decreased Ki67 proliferation; Increased mRNA and protein levels of p53 and p21^cip1/waf1^	The promotion of anti-apoptotic protein expression	2022 (68)
	*In vivo*: 10-week-old male and female C57BL/6 J mice				
	*In vitro*: HUVECs	*In vitro*: Treatment with 100 nM DOX for 24 h.	Increased SA-β-gal activity; Decreased cell proliferation; Increased protein levels of p21^cip1/waf1^ and p16^INK4a^	The ALDH1A2/AKT/ERK1/2-p21 pathway	2022 (69)
	*In vitro*: HUVECs and EA.hy926 human endothelial-derived cell line	*In vitro*: Treatment with 0.5 μM DOX for 24 h, followed by replacement with normal culture medium and monitoring after 72 and 120 h	Increased SA-β-gal activity; Increased mRNA levels of p53 and p21^cip1/waf1^	The activation of MAPK and JNK pathways-coupled with NF-κB-related oxidative stress and SASP	2023 (70)
	*In vitro*: H9c2 cells; *In vivo*: 8-week-old C57BL/6 female mice	*In vitro*: Treatment with 0.1 μM DOX for 6 days; *In vivo*: Intraperitoneal injections at 3 mg/kg, once daily for 7 days, with a cumulative dose of 21 mg/kg, collect samples for monitoring on the 9th day	Increased SA-β-gal activity; Decreased cell viability; Increased mRNA levels of p53 and p16^INK4a^; Increased expression of SASP; Elevated intracellular ROS	The promotion of mTOR protein phosphorylation	2023 (71)
	*In vitro*: Isolated neonatal mouse cardiomyocytes (NMCM); *In vivo*: 6–8-week-old ICR mice	*In vitro*: Treatment with 1 μM DOX for 72 h; *In vivo*: Treat ICR mice with intraperitoneal injections of DOX at 3 mg/kg, 6 times within two weeks, with a total cumulative dose of 18 mg/kg, collect samples on the 35th day	Increased SA-β-gal activity; Increased mRNA and protein levels of p21^cip1/waf1^ and p16^INK4a^; Increased mitochondrial fragmentation	The activation of the VPO1/ERK pathway-triggering enhanced mitochondrial fission	2024 (72)
	*In vitro*: AC16 cells; *In vivo*: C57BL/6 mice	*In vitro*: Treatment with 1.25 μM, 2.5 μM, and 5 μM DOX for 24 h; *In vivo*: Intraperitoneal injections at 2.5 mg/kg, three times a week for two consecutive weeks, with a cumulative dose of 15 mg/kg, collect samples for monitoring 4 months after completion of injections	Increased SA-β-gal activity; Decreased cell viability; Increased mRNA and protein levels of p27^kip1^, p16^INK4a^, and p21^cip1/waf1^; Increased expression of SASP	The suppression of CRIF1 expression and promotion of PXDN expression-facilitating mitochondrial fission and oxidative stress	2024 (73)
	*In vitro*: H9c2 cells; *In vivo*: 8-week-old male C57/Bl6 mice	*In vitro*: Pre-treatment with 1 μM DOX for 24 h; *In vivo*: Single intraperitoneal injection of 20 mg/kg DOX, collect samples for monitoring after 2 weeks	Increased SA-β-gal activity; Increase γ-H2AX foci; Increased mRNA levels of IGFBP3, p21^cip1/waf1^ and p16^INK4a^; Increased expression of SASP; Elevated intracellular ROS	The upregulation of PARP-2 expression, suppression of SIRT1 expression and activity, and activation of the FOXO1/p53 signaling pathway	2024 (74)
	*In vitro*: H9c2 cells; *In vivo*: C57BL/6 mice	*In vitro*: Treatment with 1.0 μM DOX for 0, 12, 24, and 48 h; *In vivo*: single intraperitoneal injection of 20 mg/kg, collect samples for monitoring after two weeks	Increased SA-β-gal activity; Increase γ-H2AX foci; Decreased cell viability; Increased mRNA levels of p53, p21^cip1/waf1^ and p16^INK4a^; Increased expression of SASP; Increased superoxide dismutase (SOD) activity; Elevated intracellular ROS	The depletion of SIRT6 and downregulation of PPARα	2024 (75)
	*In vivo*: C57BL/6 mice	*In vivo*: Administer a total cumulative dose of 24 mg/kg intraperitoneally in 6 injections over 6 weeks at 4 mg/kg per injection, and collect samples after 4 days	Increased mRNA levels of p53, p21^cip1/waf1^, p16^INK4a^ and p19^Arf^; Increased expression of SASP;	The activation of the p38/MAPK signaling pathway	2024 (76)
	*In vitro:* Human cardiac organoids (hCOs or hCardioids)	*In vitro*: Treatment with 0.5 μM DOX for 3 and 32 days;	Decreased expression of Ki67; Increased mRNA levels of p15, p21^cip1/waf1^, p16^INK4a^ and p19^Arf^; Elevated intracellular ROS	The activation of the oxidative stress pathway	2025 (77)
	*In vitro*: Cardiomyocytes differentiated from human induced pluripotent stem cells (iPSC); *In vivo*: C57BL/6 mice	*In vitro*: Treatment with 1.0 μM DOX for 24 h; *In vivo*: Administer a total cumulative dose of 20 mg/kg intraperitoneally in 4 injections over 4 weeks at 5 mg/kg per injection, and collect samples at the end of the 6th week	Increased SA-β-gal activity; Increased mRNA and protein levels of p53, p16^INK4a^, and p21^cip1/waf1^; Elevated mitochondrial ROS	The leakage of mtRNA and the activation of the cGAS-STING pathway	2025 (78)
Radiotherapy	*In vitro*: Bovine Aortic Endothelial Cells (BAECs) and HUVECs	*In vitro*: Sample collection 3–5 days after 8 Gy ionizing radiation	Increased SA-β-gal activity; Cellular senescence morphology; Cell cycle arrest; Increased γ-H2AX levels; Increased expression of SASP	The damage of DNA	2007 (79)
	*In vitro*: HL-1 mouse cardiomyocytes and H9c2 rat cardiomyocytes	*In vitro*: Sample collection at 72 h and 96 h post-exposure to 0, 2, and 8 Gy ionizing radiation	Increased SA-β-gal activity; Decreased cell proliferation; Cellular senescence morphology; Elevated intracellular ROS	The accumulation of ROS	2015 (80)
	*In vitro*: Human aortic endothelial cells	*In vitro*: Sample collection at 4 and 6 days post-irradiation with unknown Gy dose	Increased SA-β-gal activity; Decreased cell proliferation; Cellular senescence morphology; Increased γ-H2AX levels; Increased protein levels of p53, p21^Cip1/Waf1^, and p16^INK4a^	The increase of GDF15 expression, ROS accumulation and activation of ERK signaling pathway along with p16/Rb pathway	2016 (81)
	*In vitro*: Human cardiomyocytes (HCMs)	*In vitro*: Sample collection at 24, 48, and 72 h post-exposure to 5 Gy ionizing radiation	Increased SA-β-gal activity; Decreased cell proliferation; Increased mRNA levels of p21^Cip1/Waf1^ and p16^INK4a^; Elevated intracellular ROS	The inhibition of SIRT1, upregulation of miR-34a expression and induction of oxidative stress	2018 (82)
	*In vivo*: Carotid arteries of apolipoprotein E knockout (ApoE^−/−^) mice	*In vivo*: After 2 weeks of inducing atherosclerotic lesions by ligating the left carotid artery, perform 6Gy whole-body irradiation on mice, collect samples after 4 weeks	Increase DNA damage-related 53BP1 foci; Increased γ-H2AX levels; Increased protein levels of p16^INK4a^; Increased mRNA levels of p21^Cip1/Waf1^ and p16^INK4a^	The DNA damage-coupled with SASP and other inflammatory factors	2021 (83)

#### 3.1.1 DNA damage and telomere deficiency

DOX exerts its cardiotoxicity effects by intercalating base pairs in DNA or forming a ternary complex with topoisomerase II and DNA, which leads to DNA double-strand breaks and replication arrest—key mechanisms contributing to cardiotoxicity, cell cycle arrest, and cellular senescence (48, 84–86). Abnormalities in the DDR also play a role in DOX-induced senescence (87). Low-dose DOX activates the DDR, recruits the ataxia-telangiectasia mutated (ATM) protein, and triggers the p53-p21 pathway, ultimately inducing cellular senescence.

As telomeres shorten with each cell division, they eventually reach a critical length that triggers entry into cellular senescence (14). DOX exposure accelerated this process by aggravating telomere attrition. Telomere attrition, a progressive loss of protective caps at chromosome ends, is another key factor in DOX-induced cardiomyocyte senescence. A low dose of 0.1 µM DOX downregulates the expression of telomeric repeat-binding factor 1 (TRF1) and telomeric repeat-binding factor 2 (TRF2) through MAPK and p53-mediated pathways, impairing telomere protection and exacerbating telomere damage and cellular senescence (48, 51). Moreover, telomere attrition activates the DDR, culminating in senescence in neonatal rat cardiomyocytes. In contrast, high doses of DOX promote early cardiomyocyte apoptosis by reducing TRF2 levels and increasing TRF1 expression (49). Similar results have been observed in senescent smooth muscle cells.

#### 3.1.2 Oxidative stress

Redox reactions are central to fundamental biological processes in humans. According to the “Free Radical Theory of Aging,” aging is primarily driven by the overactivation of free radical reactions or excessive accumulation of ROS (88). The overaccumulation of ROS can damage biological macromolecules, such as lipids and proteins, leading to oxidative stress, cell senescence, and ultimately organ failure. In a DOX-induced mouse model of cardiomyopathy, cardiomyocyte senescence was linked to the upregulation of thioredoxin-interacting protein (TXNIP), which inhibited thioredoxin activity and disrupted intracellular antioxidant balance (56). DOX-treated HL-1 cardiomyocytes exhibited features of cellular senescence, including reduced mitochondrial membrane potential, increased ROS, and elevated malondialdehyde (MDA) levels, which were associated with higher expression of lincRNA-p21 and activation of the Wnt/β-catenin signaling pathway (53).

#### 3.1.3 Anti-apoptotic proteins

Apoptosis and cellular senescence represent two distinct cellular responses to damage. Senescent cells are highly resistant to apoptosis due to the upregulation of various anti-apoptotic proteins, such as members of the Bcl-2 family, Survivin, and XIAP (X-linked inhibitor of apoptosis protein) (89). In DOX-treated cardiomyocytes, the upregulation of these anti-apoptotic proteins contributes to a senescent, pro-inflammatory phenotype (90). Furthermore, activation of anti-apoptotic signaling pathways, including the PI3K/Akt pathway (90, 91), the Nrf2 pathway, and the NF-κB-IAP pathway, has also been observed in the senescence cardiomyocytes (92, 93). Clearing senescent cells by downregulating these anti-apoptotic proteins can alleviate chemotherapy-induced cardiovascular aging (66, 68). The novel senolytic agent ABT-263 (Navitoclax), which targeted the Bcl-2 family, effectively induced apoptosis in DOX-induced senescent cardiomyocytes and primary human umbilical vein endothelial cells (HUVECs) (68). However, ABT-263 failed to induce apoptosis in senescent somatic hybrid cells, likely due to differences in the levels of anti-apoptotic proteins (66).

#### 3.1.4 Protein homeostasis

Protein homeostasis is essential for maintaining cellular structure and function, preventing the accumulation of metabolic waste, and playing a pivotal role in cardiovascular aging and cardiac myocyte renewal. Autophagy, the lysosomal degradation of misfolded and aggregated proteins as well as damaged organelles, is a key process in protein homeostasis. Therefore, autophagy regulates cardiovascular aging and the renewal of cardiac myocytes by maintaining protein homeostasis. In DOX-treated cardiomyocytes, a loss of autophagy-related gene 4a (ATG4a), a mediator of autophagosome formation and maturation, triggered cardiomyocyte senescence (58). The senescence induced by DOX treatment can be alleviated by exosomes derived from mesenchymal stem cells (MSCs) that express LncRNA-MALAT1^+^. This effect is mediated by inhibiting miR-92a-3p and promoting ATG4a expression and autophagosome formation. AMPK, a key kinase involved in energy homeostasis and a known positive regulator of autophagy, plays a key role in regulating cardiomyocyte senescence. Activation of AMPK can delay or prevent cancer therapy-related cardiovascular senescence and diseases. Yusei Fujioka et al. found that DOX-induced senescent fibroblasts increased the secretion of extracellular vesicles (EVs) with characteristics of autophagy activation, possibly through AMPKα activation (94). However, whether autophagy induced by EVs is protective or exacerbates cardiac pathology requires further investigation.

#### 3.1.5 Epigenetic modifications

Epigenetic alterations, such as modifications in the genome, heterochromatin disruption, DNA methylation patterns, and histone modifications (e.g., histone acetylation), are critical for gene expression regulation. Loss of epigenetic information has been demonstrated in progeroid mice (8, 95). Compared to newborns, aged populations exhibit lower DNA methylation levels, with a 7% reduction in the methylation status of neighboring cytosine—phosphate—guanine (CpG) sites (96, 97). DOX has been shown to alter the metabolism of S-adenosylmethionine (SAM), a key methylation donor, and decrease global DNA m5C levels in cardiac tissue (52). Given the role of DNA methylation in senescence, it is plausible to speculate that DNA methylation plays a role in DOX-induced cardiovascular senescence.

In addition to DNA methylation, histone acetylation has recently been implicated in the development of cardiovascular aging (98). A decline in histone acetylation alters chromatin structure, which may disrupt the binding of transcription factors and repair enzymes to DNA, interfering with the normal cell cycle and promoting cellular senescence (99). In DOX-treated rats, an upregulation of histone deacetylases (HDACs) and downregulation of histone acetylation regulatory genes, such as CBP and Ep300, were observed (52, 100). Together, these alterations in DNA methylation and histone acetylation play a pivotal role in cardiovascular aging. Targeting these specific epigenetic modifications may offer a novel strategy for mitigating cytotoxic drug-induced senescence.

#### 3.1.6 Senescence-associated secretory phenotype (SASP)

Senescent cells typically exhibit a distinctive secretion profile, including various proinflammatory cytokines, chemokines, growth factors, and proteases, collectively referred to as SASP. The SASP both results from and drives cellular senescence. DOX-induced senescent cardiomyocytes secrete SASP factors that contribute to chronic low-grade inflammation, disrupting cardiac structure and function (70). Furthermore, inflammation-induced SASP can exacerbate DNA and telomere damage, while directly influencing cell cycle arrest. Key inflammatory regulators, including NF-κB, p38/MAPK, and C/EBPβ, are activated and promote SASP secretion in DOX-treated mice and cells (101–103). Additionally, SASP factors secreted by DOX-induced senescent cardiomyocytes can propagate senescence in neighboring cells through exosomal delivery (103). The oxidative stress associated with SASP factors may also be driven by the NOD-like receptor family pyrin domain containing 3 (NLRP3) inflammasome (104). The SASP is diverse, with both pro-inflammatory and anti-inflammatory factors potentially arising from the same stressor. Analyzing, evaluating, and strategically targeting the elimination of SASP represents a critical approach to alleviate the burden of cardiac aging.

In addition to the mechanisms mentioned earlier, alterations in mitochondrial structure, quantity, and function are also critical contributors to cardiac aging. Mitochondria, the primary energy-producing organelles, play an essential role in DOX-induced cardiovascular senescence (85, 105). DOX treatment disrupts mitochondrial quality control (MQC) and suppresses mitochondrial biogenesis through the peroxisome proliferator-activated receptor gamma co-activator 1-Alpha/Beta (PGC-1α/β) pathway (106), leading to a reduction in mitochondrial DNA and insufficient ATP production in the heart (52), thereby promoting the senescence phenotype (107, 108). Notably, DOX induces mitochondrial membrane destabilization via the activation of BAX/BAK-mediated mitochondrial permeability transition pores (mPTPs), where VDAC serves as a critical component. This cascade leads to the extrusion of mtDNA into the cytosol, thereby triggering the cGAS-STING inflammatory axis and driving cardiomyocyte senescence (78). The processes of mitochondrial fusion and fission are also vital in maintaining mitochondrial ROS homeostasis (109). A decrease in fusion coupled with an increase in fission promotes excessive ROS accumulation in cells, accelerating cellular senescence (73). Prolonged low-dose DOX treatment recruits dynamin-related protein 1 (DRP1) via overactivated ERK, which interacts with fission proteins such as Fis1 and mitochondrial fission factor (MFF) on the outer mitochondrial membrane, leading to exacerbated mitochondrial fragmentation in cardiomyocytes. This fragmentation causes electron leakage and excessive ROS accumulation, which further induces cardiomyocyte senescence (72). Mitochondrial autophagy, a key aspect of MQC, plays a critical role in removing damaged mitochondria. This process involves the PTEN-induced putative kinase 1 (PINK1) and the E3 ubiquitin ligase Parkin. Parkin has been shown to mitigate DOX-induced cardiac aging by promoting K63-linked polyubiquitination of TBK1, activating the TBK1/P62 signaling pathway, and facilitating type II mitophagy (60, 110).

### 3.2 Mechanisms of other chemotherapeutic agents-induced cardiovascular aging

Various chemotherapeutic agents, including platinum-based drugs, taxanes, antimetabolites and alkylating agents, contribute to treatment-induced senescence. Cisplatin can inhibit the CaMKKβ/AMPK signaling pathway and suppresses Sirtuin 3 (SIRT3) expression. As a key mitochondrial deacetylase, SIRT3 inhibition can lead to ROS accumulation and induces senescence in renal tubular cells (111). Paclitaxel, a microtubule-stabilizing agent, is known to induce cell cycle arrest and cause premature senescence in MSCs (112). Yet, Macías et al. reported that paclitaxel ameliorated abnormal heart rate variability and sinoatrial block in mice with Hutchinson-Gilford Progeria Syndrome (HGPS) (113). This indicates that cell type, disease background and other variables can be used in the study of paclitaxel in the field of aging, and its effect and mechanism of aging in the cardiovascular system warrant further investigation.

5-fluorouracil (5-FU) inhibits the expression of nitric oxide synthase (eNOS) and Sirtuin 1 (SIRT1) by activating the p38/JNK pathway, inducing endothelial senescence and dysfunction. Conversely, glucagon-like peptide-1 (GLP-1) alleviates 5-FU-induced endothelial senescence by moderately activating ERK1/2, PI3K, and PKA pathways (114). Alkylating agents, such as cyclophosphamide, ifosfamide, and busulfan, may induce premature ovarian cellular senescence by inhibiting the TrkB/Akt/ERK axis (115). The activation of ERK1/2 in the aging heart is typically impaired; Research have suggested that transduction of constitutively active MEK (CaMEK) can activate ERK1/2 signaling, inhibit mitochondrial fragmentation, and thereby alleviate ischemic damage in the aging heart (116). All the above studies indicate that balancing the MEK/ERK pathway may play an important role in protecting cellular functions, maintaining mitochondrial homeostasis, and delaying CTR-CVA (117, 118). Chen et al. demonstrated in a chronic rhinosinusitis (CRS) model that MEK1/2 and WNT2B signaling can synergistically promote mucosal repair and epithelial remodeling (119). The core logic of “MEK/WNT synergistic regulation of tissue repair” may provide a crucial regulatory node for improving tumor therapy-induced vascular endothelial dysfunction and CTR-CVA. Additionally, the impact of numerous other drugs on cardiovascular aging, including tyrosine kinase inhibitors (TKIs) and immune checkpoint inhibitors, remains insufficiently explored.

### 3.3 Mechanisms of radiotherapy-induced cardiovascular aging

Approximately 50% of patients with cancers undergo radiation therapy (120), including those with advanced nasopharyngeal carcinoma, lung cancer, liver cancer, esophageal cancer, and breast cancer (121, 122). Similar to DOX, high-energy ionizing radiation directly damages DNA and telomeres, leading to genomic instability, oxidative stress, and cellular senescence. Consequently, senescence plays a detrimental role in the onset and progression of radiation-induced cardiotoxicity (RIC). Endothelial cells, key components of the cardiovascular system, are particularly prone to senescence or permanent cell-cycle arrest after exposure to moderate or high radiation doses (123). These cells are critical initiators of RIC and are highly susceptible to radiation-induced senescence (124–126). Park et al. demonstrated that overexpression of GDF15 in human aortic endothelial cells (HAECs) promoted ROS production, overactivated the ERK signaling pathway, and induced the cellular senescence via the p16^INK4a^-Rb pathway following ionizing radiation exposure (81).

In addition to direct radiation-induced damage, the radiation-induced by-stander effect (RIBE) also plays a significant role in cellular senescence (127, 128). The ROS and SASP factors released by irradiated cells can cause oxidative damage and chronic inflammation in neighboring cells. Zhang et al. observed a shift from an acute stress-associated phenotype (ASAP) to SASP in stromal cells exposed to radiation (129). Various cytokines and chemokines, including interleukins, TNF-α, and TGF-β, are secreted by irradiated cells under radiation stress and can affect adjacent non-irradiated cells via gap junctions, paracrine signaling, or exosome-mediated communication (128, 130). Consequently, the bystander effect has significant implications for radiation-induced cardiovascular aging.

## 4 Monitoring of cancer treatment-related cellular senescence and cardiovascular aging

Cardiovascular aging and cellular senescence are primarily monitored through biomarkers, histological analysis, and functional evaluations. Non-invasive tracking of cardiovascular aging and cellular senescence is essential for diagnosis and predicting disease progression. Currently, most non-invasive tracking senescence indicators are indirect evidence, such as using cine-MRI to assess arterial strain as a surrogate for vascular aging. Common imaging indicators for monitoring cardiovascular aging include myocardial mass, tissue composition, ventricular volumes, systolic and diastolic function, left ventricular (LV) strain, and left atrial volumes and function (131). The following sections will highlight the latest research in three key areas of cardiovascular aging monitoring: cytological, functional, and histological assessments.

### 4.1 Biomarker-based cancer treatment-related cardiovascular aging monitoring

Imaging and monitoring of senescent cells have gained significant attention in recent years, with the development of simplified tools for effective detection. The combination of cellular senescence biomarkers with optical or imaging techniques has enabled the creation of specific tracers for real-time monitoring of senescent cell distribution and dynamic changes in living organisms, providing more intuitive guidance for clinical applications. Beyond ex vivo analyses, such as blood or omics studies, the primary *in vivo* monitoring tools for cellular senescence are largely based on biomarkers like SA-β-gal (132), ROS, and p16^INK4a^, often using PET or MRI imaging. However, while these tools excel at detecting senescent cancer cells, their application for monitoring cardiovascular senescence remains limited.

#### 4.1.1 Monitoring tools based on β-galactosidase (SA-β-gal)

β-Galactosidase is a classic biomarker for cellular senescence monitoring. β-Gal-based probes typically consist of two key components: a recognition group (β-galactoside) and a fluorescent reporting group. Upon hydrolysis by the elevated β-galactosidase in senescent cells, the probe is activated, emitting fluorescence.

Among various materials, aggregation-induced emission (AIE) materials are distinguished by their exceptional photostability and sensitivity to aggregation environments. Cen et al. developed the QM-β-gal probe, incorporating an AIE luminogen (AIEgen), which effectively detects DOX-induced senescent cancer cells (132). Notably, the QM-β-gal probe maintains stable *in vivo* imaging for up to 14 days post-injection, offering significant potential for monitoring cancer cell recurrence and metastasis. However, the probe was unable to detect cellular senescence in the heart, likely due to the lower cumulative dose of DOX compared to conventional doses used in cardiovascular aging models, coupled with a relatively short tissue sampling time (132). Recently, the β-Gal-targeting PET imaging probe, ^68^Ga-BGal, was designed to directly monitor cellular senescence in live animals, highlighting its considerable clinical translation potential (133).

Notably, not all β-galactosidase-positive cells are necessarily senescent, indicating that false-positive results must be considered in clinical practice.

#### 4.1.2 Monitoring tools based on reactive oxygen species

Excessive accumulation of hypochlorous acid (HClO) is a major contributor to elevated intracellular ROS levels, which serve as a hallmark of senescent cells. Consequently, HClO represents a promising target for monitoring cellular senescence. Li et al. developed a novel probe to detect HClO levels both *in vitro* and *in vivo*, which can effectively differentiate senescent cells from non-senescent ones in cardiac tissues. The near-infrared fluorescent probe SA-HCy-1 responds to three key indicators—SA-β-gal, high ROS levels, and elevated lysosomal pH—enabling the visualization of short-term senescence in live cells within a skin photoaging mouse model (134). However, its clinical applicability requires further investigation, particularly regarding its correlation with classical markers, such as p21^cip1^ and p16^INK4a^.

#### 4.1.3 Other monitoring tools

Radioactive tracers targeting p16^INK4a^ allow for *in vivo* imaging of cellular senescence via positron emission tomography (PET) (135). Monitoring telomerase activity and the expression of human telomerase reverse transcriptase (hTERT) in CD34(+) circulating progenitor cells has emerged as a simple and effective method for assessing cardiac aging (136). Moreover, SASP expression in plasma has been more strongly linked to cardiovascular aging, suggesting SASPs as potential biomarkers for monitoring this process (137).

### 4.2 Function-based cancer treatment-related cardiovascular aging monitoring

Cardiovascular aging and cellular senescence are typically associated with functional changes, including reduced cardiac contractility, impaired diastolic function, decreased vascular elasticity, and abnormal cardiac conduction. Functional monitoring indicators for cardiovascular aging include left ventricular ejection fraction (LVEF), the E/A ratio, global longitudinal strain (GLS), and pulse-wave velocity (PWV) (16). Selecting appropriate and accessible functional monitoring metrics will aid in the indirect diagnosis and early prediction of cancer treatment-related cardiovascular aging.

#### 4.2.1 Monitoring of cardiac contraction

Impaired cardiac contractility during cancer treatment is primarily attributed to factors such as cardiomyocyte loss, myocardial microvascular remodeling, reduced coronary perfusion, and collagen deposition in the myocardium. The conventional quantitative parameter for assessing cardiac contractile function is LVEF, which commonly declines in the early stages of cancer treatment-related cardiovascular aging (138). Thus, traditional CTR-CVT LVEF data can serve as a useful benchmark. Three-dimensional transthoracic echocardiography (3D TTE) is the preferred method for LVEF evaluation. In cases where 3D echocardiography is not feasible, the modified two-dimensional (2D) Simpson's biplane method can be used as an alternative (139, 140). Notably, cancer treatment-related cardiovascular aging often occurs with preserved ejection fraction. Therefore, a normal LVEF value cannot rule out cardiac contractility dysfunction.

#### 4.2.2 Monitoring of diastole

Cancer treatment induces cardiac fibroblast transformation into myofibroblasts, which secrete excessive collagen, leading to cardiac fibrosis. This fibrosis alters the mechanical properties of the heart chambers, increasing stiffness and reducing elasticity (141). Additionally, cancer therapies can impair calcium transport proteins and disrupt calcium homeostasis in cardiomyocytes, contributing to diastolic dysfunction. Diastolic function indicators are therefore valuable for indirectly assessing cancer treatment-related cardiovascular aging. Early-stage DOX-induced cardiomyopathy is primarily characterized by diastolic dysfunction (142, 143). Radiotherapy can also cause diastolic dysfunction, often linked to cardiomyocyte damage, microvascular occlusion, and ECM remodeling. Additionally, DOX treatment induces collagen deposition in the atrial walls, leading to increased stiffness, which can be indexed by the diastolic capacity of the left atrium (LA). This suggests that LA diastolic capacity may also serve as a indicator for cancer treatment-related cardiovascular aging (144, 145). Magnetic resonance radiomics, based on major and minor axis lengths and 2D diameter imaging, has revealed a reduction in LA chamber size that is strongly associated with cardiovascular aging, highlighting the potential value of magnetic resonance radiomics in monitoring cancer treatment-related cardiovascular aging (146).

#### 4.2.3 Monitoring of strain

Myocardial strain capacity is influenced by muscle length and angles, encompassing longitudinal, radial, and circumferential dimensions. A decline in myocardial strain capacity is a hallmark of aging cardiovascular systems, suggesting that strain capacity also serves as a functional indicator of cancer treatment-related cardiovascular aging. Key quantitative imaging indicators for myocardial strain capacity include global longitudinal strain (GLS), global circumferential strain (GCS), global radial strain (GRS), left ventricular twist (LV Twist), and Torsion (146). GLS, in particular, is recommended for evaluating left ventricular strain in CTR-CVD and can be used to indirectly monitor cancer treatment-related cardiovascular aging (147).

#### 4.2.4 Monitoring of conduction

Dysfunction of the cardiac conduction system is associated with a range of physiological and pathological cardiovascular changes (148, 149). Cancer treatment-related cardiovascular aging exhibits similar characteristics to general organ aging (29), including a reduction in heart rate and QT interval prolongation (150, 151). Baseline electrocardiogram (ECG) monitoring prior to treatment, incorporating the Fridericia correction method (QTcF) (152), along with dynamic ECG (Holter monitoring), are common tools for tracking transient arrhythmias and conduction abnormalities.

The integration of AI, wearable devices, electronic stethoscopes, and ECG technology (153) has enabled the application of deep learning models to more precisely assess conduction system dysfunction, predict cancer treatment-related cardiovascular aging, and guide drug administration (154, 155). Significant alterations in the QT interval, exceeding established thresholds, signal the need to promptly discontinue non-essential medications during cancer treatment-related cardiovascular aging (150, 156, 157). However, it is important to recognize that current machine learning models are limited by data constraints, and the performance of external applications still requires optimization.

### 4.3 Histology-based cancer treatment-related cardiovascular aging monitoring

Histological monitoring offers more direct structural insights into CTR-CVA, facilitating dynamic evaluations. Given the high risks associated with pathological biopsy, current histological indicators primarily rely on imaging parameters to indirectly assess myocardial mass, cardiac fibrosis, myocardial perfusion, coronary artery calcification (CAC), and valvular calcification.

High levels of oxidative stress and chronic inflammation are potential mechanisms driving vascular aging associated with cancer therapies (158). CMR first-pass perfusion imaging, which assesses myocardial perfusion index (PI), may reveal alterations in cardiac microcirculation in patients undergoing prolonged chemotherapy, serving as a valuable reference for CTR-CVA evaluation (159). Radionuclide myocardial perfusion imaging, reflecting myocardial perfusion via radiolabeled compound uptake by myocardial cells, could also become a useful tool for CTR-CVA assessment. Additionally, automated coronary calcium scoring from CT scans offers significant advantages in evaluating the extent of coronary aging in patients with cancers. PET/CT has been shown to effectively monitor CAC (160, 161), though clinical studies specifically addressing CAC resulting from both aging and cancer treatment remain scarce. Certain chemotherapeutic agents, such as alkylating drugs, may induce ovarian insufficiency, leading to decreased estrogen levels (162). Therefore, changes in estrogen levels must be considered when monitoring arterial stiffness in patients undergoing cancer treatments. Addressing these challenges requires personalized assessment, monitoring, and follow-up to optimize patient care.

Given these indicators, refining conventional monitoring methods for CTR-CVA is essential. Establishing a comprehensive baseline for cardiovascular aging is critical. More high-quality randomized controlled trials (RCTs) should be prioritized, and long-term follow-up assessment protocols should be systematically adopted to achieve a more comprehensive, objective, and accurate comprehensive evaluation of CTR-CVA. Developing a non-invasive imaging system capable of monitoring aging from cellular senescence to histological and functional changes could significantly enhance clinicians' ability to detect early cardiovascular aging in patients and foster more precise interventions, ultimately improving survival outcomes.

## 5 Treatment of cancer treatment-induced cardiovascular aging

Cellular senescence is an important influencing factor of CTR-CVT. Therefore, anti-aging interventions may mitigate the progression of CTR-CVT and CTR-CVA. Current strategies for preventing and treating cardiovascular aging can be divided into pharmacological and non-pharmacological approaches. Currently, the prevention and management of CTR-CVA remain challenging.

### 5.1 Pharmacotherapy

Anti-aging drugs, or senotherapeutics, are designed to target both intracellular senescence-associated pathways and extracellular membrane proteins specifically upregulated in senescent cells. These agents are categorized into senolytics and senomorphics. Senolytics selectively eliminate senescent cells, whereas senomorphics modulate the SASP without inducing cell death. Immunotherapy, an emerging approach, aims to specifically eliminate senescent cells by targeting unique surface markers (Table 3).

**Table 3:** Applications of various anti-aging therapies in the cardiovascular field (2015–2025).

Classification	Name	Experimental model	Treatment protocols	Literature results in detail	Year
Senolytics	Dasatinib + Quercetin (D + Q)	*In vivo*: 1)6-week-old C57BL/6 mouse diabetes model; 2)Cardiac tissue and cardiac stem cells (CSCs) from clinical non-elderly type 2 diabetes mellitus (T2DM) patients; *In vitro*: High-glucose model of CSCs	*In vivo*: After T2DM model establishment, intraperitoneal injection of 5 mg/kg D and 50 mg/kg Q for 3 consecutive days, repeated for 4 weeks, then sampling.	D + Q eliminated cardiac senescent cells (including CSCs), restored CSC function, promoted cardiomyocyte regeneration, reduced myocardial fibrosis, and improved diabetes-related cardiac diastolic function.	2022 (163)
			*In vitro*: After high glucose-induced senescence, treated with 0.25 μmol/L D and 10 μmol/L Q, repeated every 2 days for 4 days, sampling 2 days after the last treatment		
		*In vivo*: 22–24-month-old female C57BL/6 mouse myocardial infarction (MI) model	*In vivo*: Intraperitoneal injection of 5 mg/kg D and 50 mg/kg Q for 3 consecutive days weekly starting after MI, sampling after 4 weeks	D + Q eliminated senescent cells, improved the inflammatory microenvironment, and activated cardiac stem cell function, which significantly enhanced cardiac function after myocardial infarction in aged female mice.	2022 (164)
		*In vitro*: a Human Model of Cardiac Fibrosis-on-a-Chip	*In vitro*: After 1 week of microarray construction, treated with 0.1 μM D and 2 μM Q for 2 weeks under continuous TGF-β stimulation, sampling after treatment	D + Q selectively induced apoptosis of senescent myofibroblasts, significantly improved systolic function, and reduced passive tension, with effects superior to traditional antifibrotic drugs (such as pirfenidone and losartan).	2023 (165)
		*In vivo*: 2-month-old rat MI model	*In vivo*: Gavage of 5 mg/kg D and 50 mg/kg Q starting 4 h after MI, once daily for 3 consecutive days, repeated after a 2-week interval, then sampling	D + Q significantly reduced the proportion of p16^+^αSMA^+^ myofibroblasts and p16^+^CD31^+^ endothelial cells in the atria of MI rats, reversed atrial remodeling, and decreased the atrial fibrillation induction rate (from 89% to 0%).	2024 (166)
		*In vivo*: 8-week-old female C57BL/6NTac mouse obesity model	*In vivo*: After obesity model establishment (week 16), gavage of 5 mg/kg D and 50 mg/kg Q for 3 consecutive days weekly until sampling at week 24	D + Q reduced SA-β-Gal^+^ cells in cardiac tissue, decreased the protein expression of p16/p21/p53, and improved obesity-related cardiac dysfunction.	2024 (167)
		*In vivo*: 1) 22–24-month-old C57BL/6 mouse aging model; 2) Various disease models: MI, heart failure (HF), hypertension, atherosclerosis, abdominal aortic aneurysm (AAA).	*In vivo*: Intraperitoneal injection of 5 mg/kg D and 50 mg/kg Q 3 days weekly for 2–4 weeks after model establishment, sampling 24–48 h after last administration or 4–8 weeks after model induction	D + Q eliminated senescent cells in various models, improved function, and improved the microenvironment.	2024 (168)
		*In vitro*: DOX-induced senescence model of human induced pluripotent stem cell (hiPSC)-derived cardiac organoids (hCardioids)	*In vitro*: After establishing DOX-induced senescent cardiac organoid model, treated with 0.25 μM D and 2 μM Q for 2 weeks under continuous TGF-β stimulation, sampling after treatment	D + Q eliminated senescent cells, promoted recovery of progenitor cell function, inhibited fibrosis, and effectively reversed DOX-induced senescent cardiomyopathy.	2025 (77)
	Navitoclax (ABT263)	*In vivo*: 3–4-month-old male C57BL/6J mouse myocardial ischemia-reperfusion injury (IRI) model	*In vivo*: Gavage of 50 mg/kg Navitoclax on days 4–10 after model establishment, sampling after five weeks	Navitoclax and its targeted formulations eliminated DOX-induced senescent cardiomyocytes, alleviated cardiotoxicity, and restored cardiac function.	2020 (169)
		*In vivo*: 6–8-week-old male C57BL/6J mouse AngII-induced senescence model; *In vitro*: Primary cardiomyocytes and fibroblasts AngII-induced senescence models	*In vivo*: Gavage of 50 mg/kg Navitoclax for 2 cycles (7 days per cycle, 2-week interval), sampling 1 week after last administration; *In vitro*: Treatment with 1 µmol/L Navitoclax for 72 h after model establishment, then sampling	Navitoclax eliminated AngII-induced senescent cardiomyocytes and fibroblasts, inhibited SASP, inflammation, and fibrosis, and improved cardiac function and electrophysiological properties.	2020 (170)
		*In vivo*: 10-week-old C57BL/6J mouse DOX-induced senescence model (chronic low-dose multiple administration); *In vitro*: HL-1 cardiomyocyte line DOX-induced senescence model	*In vivo*: Intraperitoneal injection or gavage of three Navitoclax formulations after model establishment, sampling 30 days post-last administration *In vitro*: Treatment with 0.37 µM Navitoclax for 48 h after model establishment, then sampling	Navitoclax and its targeted formulations eliminated DOX-induced senescent cardiomyocytes, alleviated cardiotoxicity, and restored cardiac function.	2022 (68)
		*In vitro*: DOX-induced senescence models in EA.hy926 cells and HUVECs	*In vitro*: Treatment with 0.1–10 uM ABT-263 for 24–48 h after model establishment, then sampling	ABT-263 selectively eliminated senescent HUVECs by inhibiting anti-apoptotic proteins such as BCL-xL.	2022 (66)
		*In vivo*: 6-month-old *Atg*7cKO mouse autophagic deficiency model;	*In vivo*: Gavage of 50 mg/kg Navitoclax for 2 cycles (5 days per cycle, 1-week interval) after model establishment, sampling 1 week post-last administration	Navitoclax improved the function of autophagy-deficient hearts by eliminating senescent cells.	2024 (171)
		*In vivo*: Male Sprague-Dawley (SD) rat abdominal aortic constriction (AAC)-induced HF model; *In vitro*: Neonatal rat cardiomyocytes (NRCMs) and mouse H9c2 cardiomyocyte line AngII-induced senescence models	*In vivo*: Intravenous injection of 25 mg/kg ABT263 and equivalent dose of AP@ABT263 nanoassemblies at weeks 9, 12, 15, 18 after model establishment, sampling 1 week post-last administration; *In vitro*: Treatment with 5 μM ABT263 and equivalent dose of AP@ABT263 for 48–72 h after model establishment, then sampling	AP@ABT263 achieved precise elimination of senescent cardiomyocytes, significantly improved cardiac function in the HF model, and avoided the systemic toxicity of free drugs.	2024 (172)
	Fisetin	*In vivo*: 1) Ercc1^−/*Δ*^ progeria mice; 2) 22–24-month-old C57BL/6 mice; *In vitro*: Oxidative stress-induced senescence model in mouse embryonic fibroblasts (MEFs); Etoposide-induced senescence model in human fibroblasts (IMR90)	*In vivo*: 1) Ercc1^−/*Δ*^ progeria mice intermittently fed with 500 ppm fisetin (≈60 mg/kg) diet at 6–8 and 12–14 weeks of age, sampling 1–2 weeks post-last intervention; 2) 22–24-month-old C57BL/6 mice gavaged with 100 mg/kg fisetin for 5 consecutive days, sampling 3 days after drug withdrawal; 3) C57BL/6 mice fed with 500 ppm fisetin diet starting at 85 weeks of age until natural death or 24 months, then sampling; *In vitro*: Treatment with 1–15 μM fisetin for 24–48 h after model establishment, then sampling	The natural flavonoid fisetin exhibited highly efficient senolytic activity, improved cardiac ejection fraction in aged mice, increased vascular reactivity, and prolonged lifespan.	2018 (173)
		*In vivo*: 66-week-old aged mice; *In vitro*: DOX-induced senescence model in rat thoracic aortic smooth muscle cells (VSMCs)	*In vivo*: Gavage of 50 mg/kg fisetin for 4 consecutive weeks after model establishment, sampling on day 28; *In vitro*: Treatment with 10/50 μM fisetin for 6 h after model establishment, immediate sampling	Fisetin reversed VSMCs senescence via the PTEN/mTORC2-Akt (Ser473) signaling pathway.	2021 (174)
		*In vivo*: DOX-induced senescence model in 7-week-old male C57BL/6 mice; *In vitro*: H₂O₂-induced senescence model in rat primary VSMCs	*In vivo*: Gavage of 50 mg/kg fisetin once daily for 4 weeks after model establishment, then sampling; *In vitro*: Treatment with 10–100 μM fisetin for 12 h after model establishment, immediate sampling	Fisetin alleviated oxidative stress-induced VSMCs senescence by activating PPARγ and regulating the mTORC2-FoxO3a-autophagy cascade.	2023 (175)
		*In vitro*: H₂O₂-induced senescence model in rat primary VSMCs	*In vitro*: Treatment with 10/20/50 μM fisetin for 12 h after model establishment, immediate sampling	Fisetin alleviated VSMCs senescence via the PTEN-mediated PKC*δ*-NOX1 pathway.	2023 (176)
		*In vivo*: 1) 27-month-old wild-type C57BL/6N mice; 2) p16–3MR transgenic mice; *In vitro*: Replicative senescence model in HUVECs and human aortic endothelial cells (HAECs)	*In vivo*: Gavage of 100 mg/kg fisetin for 1 week, followed by 2-week drug withdrawal and another 1-week administration after model establishment, sampling 1 week post-last administration	Intermittent supplementation with fisetin reversed arterial dysfunction in aged mice by eliminating vascular senescent cells, reducing oxidative stress and inflammation, and improving arterial structure.	2024 (177)
			*In vitro*: Treatment with 0–1 μM fisetin for 48 h after model establishment, immediate sampling		
		*In vivo*: Rat arteriovenous fistula (AVF) model	*In vivo*: Intraperitoneal injection of 30 mg/kg fisetin for 3 weeks after model establishment, then sampling	Fisetin promoted outward remodeling of AVF and improved AVF blood flow by blocking heme-related pro-senescence effects.	2024 (178)
		*In vivo*: 1) C57BL/6 mouse T2DM model; 2) 16-month-old C57BL/6 mice; *In vitro*: High glucose-induced senescence model in HUVECs and VSMCs	*In vivo*: Gavage of 50/100/200 mg/kg fisetin or 100 mg/kg fisetin and 100 mg/kg Metformin (Metformin daily gavage for 12 consecutive weeks, fisetin treated for 3 days every 2 weeks for 12 weeks) after model establishment, sampling 3 days post-last administration; *In vitro*: Treatment with 10–50 μM fisetin for 48–72 h after model establishment, immediate sampling	Fisetin induced apoptosis of senescent cells by targeting the Pi3k-Akt-Bcl-2/Bcl-xl pathway, reduced vascular remodeling, and alleviated aortic aging in T2DM mice.	2025 (179)
Senomorphics	Metformin	*In vitro*: High glucose-induced senescence model in HUVECs	*In vitro*: Treatment with 5 μM resveratrol and 50 μM metformin after model establishment, including whole-course treatment (3 days of high glucose and 3 days of normal glucose) or stage-specific treatment (3 days of high glucose only or 3 days of normal glucose only), sampling on day 6	Metformin and Resveratrol reversed the metabolic memory of endothelial cell senescence by activating SIRT1, regulating p53 acetylation, and resisting oxidative stress.	2015 (180)
		*In vivo*: ApoE^−/−^ mouse atherosclerosis model; *In vitro*: Replicative senescence model in HAECs	*In vivo*: 50 mg/kg metformin added to drinking water after model establishment, sampling at 14 months; *In vitro*: Continuous passage with 2 mM metformin until P15, then sampling	Metformin regulated mitochondrial biogenesis and endothelial cell senescence via AMPK-mediated H3K79 methylation.	2018 (181)
		*In vivo*: DOX-induced senescence model in male C57BL/6 mice; *In vitro*: DOX-induced senescence model in rat thoracic aortic VSMCs	*In vivo*: Gavage of 200 mg/kg metformin daily for 4 weeks after model establishment, sampling 5 days post-last administration; *In vitro*: Treatment with 2 mM metformin for 1 h after model establishment, then sampling	The Metformin alleviated VSMCs senescence by regulating the AMPKα (Ser485)-SIRT3 pathway.	2022 (182)
		*In vivo*: 1) AngII-induced senescence model in 10-week-old C57BL/6 mice; 2) 20-month-old aged C57BL/6 mice; *In vitro*: AngII-induced senescence model in mouse VSMCs; primary aortic VSMCs from elderly humans	*In vivo*: 1) Gavage of 150 mg/kg metformin daily after model establishment, sampling on day 28; 2) 20-month-old mice treated with 3 g/L metformin in drinking water, sampling at 10 months; *In vitro*: Treatment with 200 μM metformin (daily medium change) after model establishment, sampling on day 12	Metformin delayed vascular aging by enhancing autophagic flux and inhibiting SASP and VSMCs senescence.	2022 (183)
		*In vivo*: 1) 12-month-old ApoE⁻/⁻ mouse model; 2) 20-month-old C57BL/6 mice; *In vitro*: Replicative senescence model in HAECs	*In vivo*: Gavage of low-dose (2.5 mg/kg) or physiological-dose (50 mg/kg) metformin daily for 10 weeks after model establishment, then sampling; *In vitro*: Continuous passage with 2 mM metformin until passage 15, then sampling	Mito-Esc and metformin delayed vascular aging and improved atherosclerosis by activating mitochondrial function and inhibiting oxidative stress and inflammation via the AMPK-SIRT1/SIRT6 axis.	2024 (184)
		*In vivo*: Male Wistar rat diabetic cardiomyopathy model	*In vivo*: Gavage of 200 mg/kg metformin daily for 10 weeks after model establishment, then sampling	Metformin alleviated diabetic myocardial aging through multiple pathways, including regulating macrophage phenotype, enhancing Klotho and GDF-15 expression, improving immunometabolism, and inhibiting cellular senescence.	2025 (185)
	Rapamycin	*In vivo*: 22-month-old aged mice	*In vivo*: Intraperitoneal injection of 2 mg/kg rapamycin once daily for 8 weeks, then sampling	Rapamycin alleviated MIF deficiency-related cardiac aging by activating autophagy.	2016 (186)
		*In vivo*: 8–9-week-old C57BL/6 wild-type mouse atherosclerosis model; *In vitro*: Shear stress-induced senescence model in HUVECs	*In vivo*: Intraperitoneal injection of 4 mg/kg rapamycin twice (24-hour interval), sampling 4 h post-last injection; *In vitro*: Addition of 0.5 μmol/L rapamycin under low shear stress conditions, sampling after 24 h	Rapamycin mitigated the progression of atherosclerosis under high shear stress conditions by activating autophagy, restoring endothelial cell alignment, and inhibiting inflammation and senescence.	2017 (187)
		*In vitro*: DOX-induced senescence model in SD rat thoracic aortic VSMCs	*In vitro*: VSMCs pre-treated with 2 μg/mL rapamycin for 4 h, then treated with 500 nM DOX for 4 h before sampling	The mTOR inhibitor rapamycin alleviated DOX-induced senescence by activating autophagy and downregulating p53/p21/p16.	2018 (188)
		*In vitro*: Replicative senescence model in SD rat thoracic aortic VSMCs	*In vitro*: Treatment with 20 nM rapamycin for 12 h, then sampling	Rapamycin reversed cell cycle arrest and senescence phenotype in senescent VSMCs by downregulating miR-30a, relieving its inhibition on Beclin1, and activating the autophagy pathway.	2019 (189)
		*In vitro*: VSMCs isolated from 6-month-old male PPARGC1A-knockout (ppargc1a^−/−^) and SQSTM1-knockout (sqstm1^−/−^) mice	*In vivo*: Intraperitoneal injection of 2 mg/kg rapamycin twice weekly for 2–4 weeks, sampling 24–48 h after last injection	Rapamycin effectively reversed the senescence phenotype of vascular smooth muscle cells induced by PPARGC1A or SQSTM1 deficiency by activating autophagy and inhibiting the mTOR pathway.	2020 (190)
		*In vitro*: Human cardiac progenitor cells (hCPCs)	*In vitro*: Culturing with 1 nM, 10 nM, or 100 nM rapamycin (medium changed every 2 days), continuous passage beyond P17, sampling at each passage	Rapamycin significantly delayed replicative senescence of hCPCs and restored their proliferation, migration, and multilineage differentiation capacities by inhibiting the mTOR signaling pathway and activating the STAT3-PIM1 axis.	2020 (191)
		*In vivo*: 18-month-old SD rats	*In vivo*: Daily feeding with 14 mg/kg enteric-coated microencapsulated feed containing rapamycin (equivalent to 2.24 mg/kg) for 6 months, then sampling	Rapamycin significantly reduced myocardial lipofuscin accumulation, decreased the proportion of senescent cells, and delayed cardiac aging in aged rats by activating autophagy.	2021 (192)
		*In vitro*: Homocysteine-induced senescence model in rat VSMCs	*In vitro*: Pre-treatment with 10 ng/mL rapamycin for 30 min, followed by treatment with 500 μmol/L Hcy for 24 h before sampling	The autophagy activator rapamycin reversed homocysteine-induced VSMCs senescence.	2021 (193)
		*In vitro*: H₂O₂-induced senescence model in HUVECs	*In vitro*: Treatment with 182.83 ng/mL rapamycin-functionalized carbon dots (Rapa-CDs) and 200 nM free rapamycin (Rapa) for 6 h, then sampling	Rapa-CDs significantly improved the water solubility of rapamycin, enhanced ROS scavenging capacity, inhibited the mTOR pathway, and effectively suppressed HUVECs senescence.	2024 (194)
		*In vivo*: Mouse T2DM model	*In vivo*: Intraperitoneal injection of 4 mg/kg rapamycin every 2 days for 8 weeks, then sampling	Rapamycin inhibited NLRP3 signaling, alleviated immune senescence, and slowed diabetic vascular aging.	2025 (195)
		*In vitro*: Primary senescent activated valvular interstitial cells (aVICs) isolated from canine myxoid mitral valve degeneration (MMVD)	*In vitro*: Culturing with 25–300 nM rapamycin for 48–72 h, then sampling	mTOR inhibitors (such as rapamycin) alleviated MMVD by activating autophagy to degrade p21/p16.	2025 (196)
	Curcumin	*In vivo*: Diabetes model in C57BL/6 mice	*In vivo*: Gavage with 1000 mg/kg curcumin after model establishment, sampling after 14 days	Curcumin significantly improved blood flow recovery and capillary density in the hindlimb ischemia model of diabetic mice by regulating the function of endothelial progenitor cells (EPCs).	2017 (197)
		*In vivo*: 18-month-old male C57BL/6 mice; *In vitro*: Replicative senescence models of human atrial fibroblasts (HATs) and mouse atrial fibroblasts (MAFs)	*In vivo*: After model establishment, daily feeding with 50/100 mg/kg curcumin dissolved in feed for 6 months, then sampling; *In vitro*: After model establishment, treatment with 6–12 µM curcumin for 48 h, then sampling	Curcumin improved age-related atrial fibrillation (AF) by inhibiting p300.	2023 (198)
		*In vivo*: 12–18-month-old C57BL/6 mice; *In vitro*: Replicative senescence model of human atrial fibroblasts	*In vivo*: Gavage with 50 mg/kg curcumin after model establishment, sampling after 6 months; *In vitro*: Treatment with 6, 9, or 12 μM curcumin for 24–48 h after model establishment, then sampling	The p300 inhibitor curcumin reversed age-related atrial fibrosis and reduced the risk of AF.	2023 (199)
		*In vivo*: 18-month-old male C57BL/6 mice; *In vitro*: TBHP-induced senescence model in MAFs	*In vivo*: After model establishment, daily feeding with 100 mg/kg curcumin dissolved in feed for 6 months, then sampling; *In vitro*: Treatment with unknown concentration of curcumin for 48 h after model establishment, then sampling	Curcumin reduced the atrial fibrillation induction rate in senescent mice, shortened the sinus node recovery time (SNRT), and improved electrophysiological abnormalities by alleviating atrial senescence and fibrosis.	2024 (200)
	Resveratrol	*In vitro*: High glucose-induced senescence model in HUVECs	*In vitro*: Treatment with 50 μM metformin and 5 μM resveratrol after model establishment, including whole-course treatment (3 days of high glucose and 3 days of normal glucose) or stage-specific treatment (3 days of high glucose only or 3 days of normal glucose only), sampling on day 6	Metformin and Resveratrol reversed the metabolic memory of endothelial cell senescence by activating SIRT1, regulating p53 acetylation, and resisting oxidative stress.	2015 (180)
		*In vivo*: DOX-induced cardiac injury model in male senescence-accelerated mice (SAMP8)	*In vivo*: Single intraperitoneal injection of 18 mg/kg DOX on day 1, intraperitoneal injection of 20 mg/kg resveratrol on days 2–4, sampling on day 5	Resveratrol alleviated cardiotoxicity in doxorubicin treatment of aged mice by regulating the SIRT1-USP7 axis.	2015 (201)
		*In vivo*: DOX-induced cardiomyopathy model in 3-month-old female Fischer 344 rats	*In vivo*: 1) One week after model establishment, intramyocardial injection of hCPCs pre-treated with DOX and resveratrol, sampling after 3 weeks of observation; 2) Gavage of resveratrol daily at the time of model establishment, sampling after 6 weeks of continuous administration	Resveratrol reversed DOX-induced senescence and apoptosis in hCPCs and partially restored their regenerative functions by activating SIRT1, promoting p53 deacetylation, repairing the IGF-1/Akt pathway, and enhancing antioxidant capacity.	2015 (202)
		*In vivo*: MI and IRI models in 9–11-week-old male C57BL/6J mice	*In vivo*: 1) MI model: Daily oral gavage of 320 mg/kg resveratrol for 1 week before MI model establishment, medication ceased after surgery, sampling on postoperative days 3, 7, and 14; 2) IRI model: Daily oral gavage of 320 mg/kg resveratrol for 1 week before IRI model establishment, sampling after 45 min of ischemia and 24 h of reperfusion during surgery	Resveratrol inhibited cardiomyocyte senescence by activating the Sirt1/p53 pathway and alleviated inflammatory responses by inhibiting the NLRP3 inflammasome, thereby improving ischemia-hypoxia-induced myocardial injury.	2020 (203)
		*In vivo*: Model of diabetes combined with MI in male SD rats	*In vivo*: Intramuscular injection of high-glucose rat bone marrow mesenchymal stem cells (BMSCs) pre-treated with 2 μM resveratrol for 96 h at 4 sites in the myocardial infarction border zone, sampling at 1 week and 3 weeks post-operation	Resveratrol reversed BMSCs senescence and enhanced their transplantation efficacy in diabetic myocardial infarction rats by downregulating miR-34a and activating SIRT1, as manifested by improved cardiac function, reduced myocardial fibrosis, and increased angiogenesis.	2021 (204)
		*In vitro*: 1) DOX-induced senescence model in human pluripotent stem cell-derived cardiomyocytes (hPSC-CMs); 2) DOX-induced senescence model in 3D dynamic engineered heart tissues (dyn-EHTs) constructed with hPSC-CMs and cardiac fibroblasts	*In vitro*: 1) Co-treatment with 10 μmol/L resveratrol and 0.1 μmol/L DOX for 48 h each, followed by 48 h recovery, repeated for 2 cycles, sampling on day 7; 2) Co-treatment with 10 μmol/L resveratrol and 0.1 μmol/L DOX, each cycle including a 48-hour administration period and a 5-day recovery period, repeated for 4 cycles, sampling on day 28	Resveratrol inhibited the expression of senescence markers and improved mitochondrial function.	2023 (205)
	Rutin	*In vivo*: ApoE^−/−^ mouse model of atherosclerosis	*In vivo*: Daily gavage with 40 mg/kg Rutin after model establishment, sampling after 6 weeks; *In vitro*: Treatment with 50 μM Rutin for 72 h after model establishment, then sampling	Rutin inhibited premature senescence of VSMCs in diabetic mice and reduced senescent cell accumulation in plaques by anti-oxidative stress and telomere protection, thereby increasing the number of VSMCs and stabilizing atherosclerotic plaques.	2018 (206)
		*In vitro*: H₂O₂-induced senescence model in primary thoracic aortic VSMCs			
		*In vivo*: MI model in aged rats	*In vivo*: Mesenchymal stem cells pre-treated with 10–200 μM Rutin for 1 h, then induced senescence by 400 μM H₂O₂ treatment for 2–4 h, followed by intramyocardial injection and sampling at 2 and 4 weeks	Rutin and quercetin significantly reduced ROS levels in young and senescent MSCs, decreased myocardial fibrosis, and promoted angiogenesis and cardiomyocyte regeneration.	2023 (207)
	Canagliflozin	*In vitro*: High fat-induced senescence model in HUVECs	*In vitro*: Co-treatment with 0.3 mM palmitic acid (PA) and 2.5/5/10 μg/mL Canagliflozin for 24 h, then sampling	Canagliflozin delayed vascular endothelial cell senescence by binding to p38/JNK and inhibiting their phosphorylation, reducing SASP secretion and cell cycle arrest, and scavenging ROS.	2023 (208)
		*In vitro*: High fat-induced senescence model in HUVECs	*In vitro*: Co-treatment with 0.3 mM PA and 0.1/0.5 μM Canagliflozin for 24 h, then sampling	Canagliflozin delayed the senescence of vascular endothelial cells by scavenging ROS, inhibiting the ERK signaling pathway, and synergistically suppressing the ferroptosis pathway.	2024 (209)
Immune-activating Therapies	Serum chemokine CCL17	*In vivo*: AngII-induced vascular injury model in 4-month-old wild-type mice	*In vivo*: Daily intraperitoneal injection of 100 μg anti-CCL17 for 4 consecutive weeks, followed by sampling	CCL17 neutralizing antibody treatment improved vascular aging or AngII-induced vascular dysfunction, remodeling, and immune dysregulation.	2023 (210)
	Canagliflozin	*In vivo*: High-fat diet model in C57BL/6 mice	*In vivo*: Addition of 0.03% w/w Canagliflozin to diet from day 3 to week 20, sampling at day 7 or week 4	The SGLT2 inhibitor canagliflozin improved age-related phenotypic changes by negatively regulating PD-L1 expression, enhancing endogenous immune surveillance, and clearing senescent cells.	2024 (211)

#### 5.1.1 Senolytics

Senolytics primarily induce the death of senescent cells, effectively preventing their accumulation in tissues. Regimens for senolytic therapy include combinations such as dasatinib and quercetin (D + Q), navitoclax (ABT-263), fisetin, proxofim (FOXO4-DRI), and HSP90 inhibitors.

Among these, D + Q has emerged as the most promising senolytic combination for promoting the clearance of senescent cells (212, 213). D + Q facilitates senescent cell elimination by inhibiting anti-apoptotic pathways, including the Ephrin B (EFNB) serine protease inhibitors (Serpins) and the PI3K/AKT pathway (214). Wagner et al. demonstrated that D + Q effectively reverses cardiac dysfunction induced by cellular senescence in animal models (215), while Roos et al. confirmed its efficacy in improving age-related vascular dysfunction (216). Notably, different senescent cells show certain selectivity for anti-aging drugs. For instance, the advantage of dasatinib in dealing with senescent adipocytes cannot be transferred to senescent endothelial cells, which are obviously more sensitive to quercetin treatment (214).

Navitoclax (68), a BCL-2 family inhibitor, is a classic agent used to mitigate chemotherapy-induced cardiovascular cellular senescence. Despite a long research history, its clinical translation is currently unfeasible due to safety issues such as induction of acute platelet toxicity. Fisetin (173), a natural flavonoid, exhibits senolytic activity through its metabolic byproducts. FOXO4 (217) neutralizes drug-induced toxicity and eliminates senescent cells in a DOX-induced kidney aging model by modulating p53. HSP90 inhibitors, such as 17-DMAG and alvespimycin (218), affect the PI3K/AKT/mTOR senescence-related pathway, triggering senescent cell apoptosis (219).

#### 5.1.2 Senomorphics

In addition to senolytic strategies, senomorphics offer another promising approach to mitigate age-related side effects. Most senomorphics exert their effects by modulating the signaling or transcriptional regulators of the SASP, including the NF-κB, JAK-STAT, and mTOR pathways (220). Metformin, a well-established senomorphics agent, reduces SASP expression and inhibits DOX-induced endothelial cell senescence by suppressing the JNK and NF-κB pathways (70). Long-term rapamycin treatment in senescent cardiac progenitor cells (CPCs) suppresses mTOR signaling, activates the JAK-STAT3-PIM1 pathway, decreases senescence marker expression, and restores clonogenic, migratory, and differentiative potential (221). Additionally, natural compounds such as curcumin and resveratrol (a SIRT1 activator) have been shown to regulate the mTOR pathway, mitigating low-dose anthracycline-induced cardiovascular aging (222). Rutin, an antioxidant plant compound, inhibits SASP expression across various cell lineages, including IL-6, IL-8, IL-1α, IL-1β, CXCL3, MMP3, and GM-CSF (223). In summary, while senomorphics exhibit milder anti-aging effects compared to senolytics, they are associated with lower cytotoxicity and fewer side effects.

#### 5.1.3 Immune-activating therapies

Immunotherapy is an emerging strategy aimed at specifically eliminating senescent cells by targeting distinct surface markers. Under normal physiological conditions, immune cells can effectively clear senescent cells. However, in pathological or aging states, senescent cells may evade immune surveillance, hindering their clearance (224). Additionally, senescent cells actively create an immunosuppressive environment that facilitates their survival. Thus, immunotherapies targeting cellular senescence present a promising approach for treating CTR-CVA. Katsuumi G. et al. demonstrated that canagliflozin, an SGLT2 inhibitor, downregulates PD-L1, restoring immune surveillance and promoting the clearance of senescent cells (211, 225). Anti-aging vaccines have also been developed to induce specific immune memory and actively remove senescent cells. For example, the ATRQβ-001 vaccine has been shown to slow the decline in cardiac function associated with cardiovascular aging (226).

Selective neutralizing or blocking antibodies, often categorized as senomorphic agents, have emerged as another therapeutic avenue (227). Lister et al. demonstrated that an Ephrin-B2 neutralizing antibody (B11) could suppress the SASP in senescent fibroblasts, thereby mitigating pulmonary fibrosis in mice. Furthermore, serum chemokine CCL17 has been identified as a potential biomarker for diagnosing and monitoring vascular stiffness and aging. Knockout of CCL17 in mice has been shown to slow vascular remodeling and inhibit vascular aging (210). The integrin family also may provide novel targets for the prediction and intervention of CTR-CVA. Sun et al. demonstrated that integrin β4 (ITGB4) can induce celluar senescence of vascular endothelial cells by activating the H-ras/caveolin-1/AP-1 axis, thereby exacerbating endothelial dysfunction (228). In cancer therapy, the signaling of the integrin-β family (ITGB4/ITGA3/5/6) may be overactivated (117), which can lead to endothelial celluar senescence, vascular sclerosis, and myocardial dysfunction through signal transduction (229, 230). Therefore, identifying key cytokines in the context of cancer treatment and targeting them to improve the inflammatory microenvironment is critical, as this may enhance therapies aimed at addressing cardiovascular aging.

However, the low specificity of immunotherapy may lead to immune overactivation, particularly given the heterogeneity of senescent cells. Consequently, careful selection of antigens and rigorous safety testing of vaccines are essential for clinical applications. Recently, urokinase-type plasminogen activator receptor (uPAR)-specific CAR-T cells have shown promise in efficiently eliminating senescent cells in a pulmonary fibrosis mouse model, representing a potential strategy for aging-related diseases (231).

### 5.2 Non-pharmacological therapy

In addition to pharmacological interventions, supportive therapies—such as dietary modifications, physical exercise, and mental health support—are also recommended to mitigate the progression of cardiovascular aging (232).

Dietary interventions play a key role in reducing ROS production, limiting the accumulation of nuclear and mitochondrial DNA damage, and maintaining mitochondrial homeostasis. Long-term caloric restriction has been consistently linked to a reduced incidence of age-related diseases (233, 234). Intermittent fasting, caloric restriction, and glucose restriction may elevate NAD^+^ levels, potentially offering protective effects against stress-induced cardiovascular aging (235). Nevertheless, as current evidence primarily comes from preclinical studies, clinical translation should proceed with caution to avoid synergistic toxicity. Moreover, methionine restriction and ketogenic diets contribute to improved cellular metabolism and aging mitigation. Aerobic exercise training (AET) has been shown to promote myocardial ischemia recovery by preventing the reduction of circulating endothelial progenitor cells in mice treated with a combination of doxorubicin and cyclophosphamide (32). Given the similarities in the mechanisms between cancer treatment-induced cardiovascular aging and cellular senescence, the supportive therapies outlined above may offer promise in addressing cancer treatment-induced cardiovascular aging.

Additionally, insufficient sleep is a significant risk factor for accelerating cardiovascular aging (236). A quiet, safe sleep environment and adequate rest are essential to prevent cellular damage and senescence. Psychological distress, mental stress, and cognitive biases associated with cancer and its treatments also require attention and support from both society and family.

## 6 Discussion

Advancements in cancer therapy have significantly improved the survival rates of patients with cancers. However, this progress has been accompanied by an escalating risk of cardiovascular toxicities in these patients. Striking an optimal balance between minimizing cancer treatment-related cardiac events and maximizing antitumor efficacy remains a challenge. Numerous studies have highlighted cellular senescence as a critical initiator and mediator of therapy-induced late side effects. This review focuses on exploring the relationships among cellular senescence, CTR-CVA, and CTR-CVT, aiming to build a bridge between the field of Aging and the field of Cardio-Oncology.

Cellular senescence is considered as a key initiator and mediating factor of CTR-CVT. In the field of Cardio-Oncology, CTR-CVT is a broad term that encompasses various cardiac toxicities under different treatment modalities, including all the adverse effects of cancer treatments on the cardiovascular system. As one of the core subsets of CTR-CVT, CTR-CVA possesses non-negligible independent research value, which is mainly manifested in the following aspects. (i) Differences in intrinsic mechanisms. Traditional CTR-CVT mostly emphasizes the damage caused by tumor treatments to cardiovascular cells. The intrinsic mechanisms are mostly apoptosis or necrosis of cells. In contrast, CTR-CVA emphasizes the long-term maintenance of a non-lethal pathway. The intrinsic mechanism is the activation of cellular senescence and related pathway, which ultimately leads to the acceleration of tissue aging. It has a more obvious time dependence, so the latency period is longer. (ii) Differences in the focuses of monitoring and intervention. The monitoring of CTR-CVA, on the basis of that of CTR-CVT, introduces the focus on the pathology of cellular senescence and tissue aging. The key points of intervention are to reduce senescent cells, improve the senescent phenotype, and rejuvenate the cardiovascular environment. However, the precise molecular mechanisms of CTR-CVA remain unclear, and the role of senescence in some cancer drugs prone to causing cardiotoxicity (such as immunotherapeutic agents) has not been validated by basic experiments, which hinders the development of targeted therapies.

Additionally, the lack of accurate monitoring methodologies for CTR-CVA presents another major challenge. To mitigate the side effects of CTR-CVA, future research should focus on the following areas: (i) Large-cohort, multi-center studies to identify precise humoral and imaging biomarkers of cardiac aging, which would enable early prediction of CTR-CVA onset, diagnosis of its severity, and assessment of the efficacy of related treatments; (ii) The use of advanced models such as organoids and engineered mouse models to investigate the molecular mechanisms underlying the onset and progression of CTR-CVA, thereby identifying potential therapeutic targets (iii) The development of high-throughput screening technologies to identify targeted drugs for CTR-CVA treatment, enhancing treatment specificity while minimizing toxicity to normal tissues and organs. Emerging technologies like single-cell sequencing, spatial transcriptomics, and artificial intelligence are highly relevant to the rapidly evolving field of cardio-oncology, offering significant promise for both research and clinical care. Through interdisciplinary collaboration among oncologists, cardiologists, materials scientists, and engineers, we aim to integrate cancer treatment with cardiovascular aging management across clinical pathways. By combining multi-omics technologies with AI-assisted detection, this approach will enable precise diagnosis, effective prevention, and systematic management of CTR-CVA, ultimately improving long-term outcomes for patients with cancers and survivors.

## Glossary

AAA: abdominal aortic aneurysm
AAC: abdominal aortic constriction
ABT-263: Navitoclax
AET: Aerobic exercise training
AF: atrial fibrillation
AIE: aggregation-induced emission
AIEgen: AIE luminogen
ALL: acute lymphoblastic leukemia
ApoE^−/−^: apolipoprotein E knockout
ARF: alternative reading frame
ASAP: acute stress-associated phenotype
ATG4a: autophagy-related gene 4a
ATM: ataxia-telangiectasia mutated
AVF: arteriovenous fistula
aVICs: activated valvular interstitial cells
BAECs: bovine aortic endothelial cells
CAC: coronary artery calcification
CAD: coronary artery disease
CCS: Childhood cancer survivor
CEPs: circulating endothelial progenitor cells
CPCs: cardiac progenitor cells
CpG: cytosine—phosphate—guanine
CSCs: cardiac stem cells
CTR-CVA: Cancer Therapy-Related Cardiovascular Aging
CTR-CVD: Cancer Therapy-Related Cardiovascular Diseases
CTR-CVT: Cancer Therapy-Related Cardiovascular Toxicity
CVDs: Cardiovascular diseases
DDR: DNA damage response
DOX: doxorubicin
DRP1: dynamin-related protein 1
D + Q: dasatinib and quercetin
ECG: electrocardiogram
ECM: extracellular matrix
EEAA: extrinsic epigenetic age acceleration
EFNB: Ephrin B
EPCs: endothelial progenitor cells
EVs: extracellular vesicles
GCS: global circumferential strain
GEAA: grim epigenetic ege ecceleration
GLS: global longitudinal strain
GRS: global radial strain
HAECs: human aortic endothelial cells
HATs: human atrial fibroblasts
HCF: human cardiac fibroblasts
HClO: hypochlorous acid
HCMs: Human cardiomyocytes
HDACs: histone deacetylases
HF: heart failure
HGPS: Hutchinson-Gilford Progeria Syndrome
HO-1: heme oxygenase-1
HUVECs: human umbilical vein endothelial cells
hsCRP: high-sensitivity C-reactive protein
hTERT: human telomerase reverse transcriptase
iCM: cardiomyocytes differentiated from iPSC
IGF-1: insulinlike growth factor-1
IL-2: interleukin-2
IL-6: interleukin-6
IL-7: interleukin-7
IL-8: interleukin-8
IL-10: interleukin-10
IL-17a: interleukin-17a
INK-ATTAC: Inducible and Regulatable Targeted Apoptosis of Senescent Cells
iPSC: human induced pluripotent stem cells
IRI: ischemia-reperfusion injury
LA: left atrium
LV: left ventricular
LVEF: left ventricular ejection fraction
LV Twist: left ventricular twist
mPTPs: mitochondrial permeability transition pores
mtDNA: mitochondrial DNA
MACE: major adverse cardiovascular event
MAFs: mouse atrial fibroblasts
MCP1: monocyte chemoattractant protein-1
MDA: malondialdehyde
MEFs: mouse embryonic fibroblasts
MFF: mitochondrial fission factor
MI: myocardial infarction
MMVD: myxoid mitral valve degeneration
MQC: mitochondrial quality control
MSCs: mesenchymal stem cells
NLRP3: NOD-like receptor family pyrin domain containing 3
NMCM: neonatal mouse cardiomyocytes
NRCMs: Neonatal rat cardiomyocytes
PEAA: phenotypic epigenetic age acceleration
PET: positron emission tomography
PGC-1α/β: peroxisome proliferator-activated receptor gamma co-activator 1-Alpha/Beta
PI: perfusion index
PINK1: PTEN-induced putative kinase 1
PWV: pulse-wave velocity
QTc: Corrected QT interval
RIBE: radiation-induced by-stander effect
RIC: radiation-induced cardiotoxicity
ROS: reactive oxygen species
SASP: senescence-associated secretory phenotype
SD: Sprague-Dawley
Serpins: serine protease inhibitors
SIRT1: sirtuin1
SNRT: sinus node recovery time
SOD: superoxide dismutase
TKIs: tyrosine kinase inhibitors
TNF-α: tumor necrosis factor-α
TRF1: telomeric repeat-binding factor 1
TRF2: telomeric repeat-binding factor 2
TXNIP: thioredoxin-interacting protein
T2DM: type 2 diabetes mellitus
uPAR: urokinase-type plasminogen activator receptor
VEGFA: vascular endothelial growth factor A
VEGFR-2: vascular endothelial growth factor receptor-2
VSMCs: vascular smooth muscle cells
XIAP: X-linked inhibitor of apoptosis protein
2D: two-dimensional
3D TTE: three-dimensional transthoracic echocardiography
5-FU: 5-fluorouracil.
